# Circulating microRNA signatures associated with disease severity and outcome in COVID-19 patients

**DOI:** 10.3389/fimmu.2022.968991

**Published:** 2022-08-11

**Authors:** Alessandra Giannella, Silvia Riccetti, Alessandro Sinigaglia, Chiara Piubelli, Elisa Razzaboni, Piero Di Battista, Matteo Agostini, Emanuela Dal Molin, Riccardo Manganelli, Federico Gobbi, Giulio Ceolotto, Luisa Barzon

**Affiliations:** ^1^ Department of Medicine, University of Padova, Padova, Italy; ^2^ Department of Molecular Medicine, University of Padova, Padova, Italy; ^3^ Department of Infectious-Tropical Diseases and Microbiology, Istituto di Ricovero e Cura a Carattere Scientifico (IRCCS), Sacro Cuore Don Calabria Hospital, Verona, Italy; ^4^ Maternal and Child Health Department, University of Padova, Padova, Italy; ^5^ Microbiology and Virology Unit, Padova University Hospital, Padova, Italy

**Keywords:** COVID-19, microRNA, biomarkers, innate immunity, inflammation, interferon, SARS-CoV-2, RNA-sequencing

## Abstract

**Background:**

SARS-CoV-2 induces a spectrum of clinical conditions ranging from asymptomatic infection to life threatening severe disease. Host microRNAs have been involved in the cytokine storm driven by SARS-CoV-2 infection and proposed as candidate biomarkers for COVID-19.

**Methods:**

To discover signatures of circulating miRNAs associated with COVID-19, disease severity and mortality, small RNA-sequencing was performed on serum samples collected from 89 COVID-19 patients (34 severe, 29 moderate, 26 mild) at hospital admission and from 45 healthy controls (HC). To search for possible sources of miRNAs, investigation of differentially expressed (DE) miRNAs in relevant human cell types *in vitro*.

**Results:**

COVID-19 patients showed upregulation of miRNAs associated with lung disease, vascular damage and inflammation and downregulation of miRNAs that inhibit pro-inflammatory cytokines and chemokines, angiogenesis, and stress response. Compared with mild/moderate disease, patients with severe COVID-19 had a miRNA signature indicating a profound impairment of innate and adaptive immune responses, inflammation, lung fibrosis and heart failure. A subset of the DE miRNAs predicted mortality. In particular, a combination of high serum miR-22-3p and miR-21-5p, which target antiviral response genes, and low miR-224-5p and miR-155-5p, targeting pro-inflammatory factors, discriminated severe from mild/moderate COVID-19 (AUROC 0.88, 95% CI 0.80-0.95, p<0.0001), while high leukocyte count and low levels of miR-1-3p, miR-23b-3p, miR-141-3p, miR-155-5p and miR-4433b-5p predicted mortality with high sensitivity and specificity (AUROC 0.95, 95% CI 0.89-1.00, p<0.0001). *In vitro* experiments showed that some of the DE miRNAs were modulated directly by SARS-CoV-2 infection in permissive lung epithelial cells.

**Conclusions:**

We discovered circulating miRNAs associated with COVID-19 severity and mortality. The identified DE miRNAs provided clues on COVID-19 pathogenesis, highlighting signatures of impaired interferon and antiviral responses, inflammation, organ damage and cardiovascular failure as associated with severe disease and death.

## Introduction

Severe acute respiratory syndrome coronavirus 2 (SARS-CoV-2), the cause of coronavirus disease 2019 (COVID-19), induces a spectrum of clinical conditions ranging from asymptomatic infection to life threatening severe disease, characterized by respiratory failure, shock and multi-organ dysfunction requiring admission in the intensive care unit (ICU). Old age, male sex, presence of co-morbidities like hypertension, diabetes, immunosuppression, defective interferon (IFN) response and genetic predisposition, have been identified as risk factors for severe COVID-19 and associated with increased mortality (1–3).

Several studies searched for diagnostic biomarkers of severe COVID-19 and for prognostic biomarkers of ICU admission and risk of death. For example, abnormal levels of several clinical and laboratory parameters, such as renal dysfunction, elevated C reactive protein (CRP) and D-dimer levels, high serum levels of interleukin-6 (IL-6) and tumor necrosis factor-α (TNF-α) were identified as predictors of worsening outcome in COVID-19 (4, 5). Systems biological analysis identified increased plasma levels of inflammatory mediators and defects of type I IFN response as associated with severe COVID-19 (6), while single cell transcriptomics of immune cells in critically ill COVID-19 patients identified increased expression of genes involved in cell cycle regulation, cell-specific activation markers, and antibody processing within B-, T-, and NK-cell subsets in patients who survived (7).

Circulating microRNAs (miRNAs) have been proposed as candidate biomarkers for clinical conditions, such as malignancy, cardiovascular diseases, and infectious diseases (8–11), including COVID-19 (12). MiRNA are small non-coding RNA molecules of about 22 nucleotide in length, generated from the endogenous cellular mRNAs, long noncoding RNAs, and tRNAs (13). These small RNA molecules play a key role in the fine tuning of cell functions by suppression of protein synthesis from target mRNAs (14). Host miRNAs exert an important role in innate and adaptive immune cell development, especially during infections, (11) and have been involved in the cytokine storm driven by SARS-CoV-2 infection and proposed a candidate diagnostic and prognostic biomarkers in COVID-19 patients (15–24).

In this study, we analyzed by small RNA-sequencing a large cohort of COVID-19 patients at the time of hospital admission and healthy controls (HC) to identify signatures of circulating serum miRNAs associated with SARS-CoV-2 infection, disease severity and mortality. To search for possible sources of the differentially expressed (DE) serum miRNAs, we analyzed their expression in relevant human cell types *in vitro* upon SARS-CoV-2 infection and IFN type I treatment.

## Materials and methods

### Ethics statement

All subjects or their legal representatives provided written informed consent. Serum samples were collected at admission and stored in Tropica Biobank (BBMRI-eric ID: IT_1605519998080235) upon use. The study, which was conducted in accordance with the ethical principles of the Declaration of Helsinki, was approved by the local Ethics Committee (Comitato Etico per la Sperimentazione Clinica delle Province di Verona e Rovigo) on November 24, 2020 (study protocol n 63471).

### Study subjects

The study population included 89 COVID-19 patients, who were admitted at the IRCCS Sacro Cuore Don Calabria hospital, Negrar, Verona, Italy, in the period between May 2020 and December 2020. Inclusion criteria for the study were age ≥18 years and diagnosis of SARS-CoV-2 infection confirmed by molecular testing on nasopharyngeal swabs. As exclusion criteria, pregnant women were not enrolled. Disease severity was scored into mild, moderate, and severe at the time of hospital admission according to World Health Organization COVID-19 disease severity classification criteria (25). In particular, individuals who tested positive for SARS-CoV-2 using a virologic test (i.e., a nucleic acid amplification test or an antigen test) but had no symptoms consistent with COVID-19 were classified as asymptomatic or with presymptomatic infection; individuals who had any of the various signs and symptoms of COVID-19 (e.g., fever, cough, sore throat, malaise, headache, muscle pain, nausea, vomiting, diarrhea, loss of taste and smell) but who did not have shortness of breath, dyspnea, or abnormal chest imaging were classified as mild COVID-19; individuals who showed evidence of lower respiratory disease during clinical assessment or imaging and who had an oxygen saturation (SpO2) ≥94% on room air at sea level were classified as moderate COVID-19; individuals who had SpO2 <94% on room air at sea level, a ratio of arterial partial pressure of oxygen to fraction of inspired oxygen (PaO2/FiO2) <300 mm Hg, a respiratory rate >30 breaths/min, or lung infiltrates >50% were classified ad severe COVID-19; individuals who had respiratory failure, septic shock, and/or multiple organ dysfunction were classified as cases of critical COVID-19. No cases of asymptomatic infection or critical COVID-19 at the time of hospital admission were enrolled in the study. Peripheral blood samples were collected at the time of hospital admission, and before starting medications. Sera were separated from whole blood by centrifugation for 15 min at 3,000 rpm at 4°C and stored at -80°C until processing. Serum samples from 45 healthy volunteers collected before September 2019 and stored at -80°C were used as negative control group (25 females, 20 males; median age 45, range 24-76).

### Small RNA library preparation and quantification for next generation sequencing

Small RNA libraries from serum samples were obtained using QIAseq^®^ miRNA Library kit (Qiagen, Hilden, Germany), according to the manufacturer protocol. NGS Library Quality Control (QC) analysis and quantification were performed before sequencing: a) High sensitivity DNA electrophoresis by LabChip GX Touch Nucleic Acid Analyzer (PerkinElmer, Massachusetts, USA) using HT DNA 5K/RNA LABCHIP kit (D-MARK Biosciences, Toronto, Canada) according to the manufacturer’s instructions. We obtained typical electropherograms from small RNA libraries that show a peak between 170-180 bp corresponding to miRNA-sized library; b) quantitative polymerase chain reaction (qPCR) according to the manufacturer’s protocol, using three different primers provided by QIAseq^®^ miRNA Library kit (Qiagen): the first, called NGS 3C Primer, for assessing the performance of 3’ adapter ligation; the second, NGS 5C Primer, for assessing the performance of 5’ adapter ligation and the third, NGS RTC Primer, for the performance of reverse transcription reaction. We obtained a value of threshold cycle (CT) less than 28 indicating all these steps were performed correctly. NGS library concentration was determined by Qubit dsDNA HS assay (Thermo Fisher Scientific) by Qubit^®^ 4.0 Fluorimeter, according to the manufacturer’s protocol.

### Small RNA library sequencing

Equimolar amounts (1 nM) of pooled libraries normalized to 10 nM were generated to sequence in multiplexing. PhiX DNA 1.5 pM was added to pooled libraries prior to sequencing at a final concentration of 10% in order to increase the sequence diversity of the libraries. Pooled small RNA libraries (1.7 pM) were sequenced using NextSeq™ 550 System (Illumina, San Diego, California, USA) following manufacturer’s instructions. NextSeq 500/550 High Output Kit v2.5 (75 Cycles) was used for sequencing in single reads of 75 pb fragments for small RNA library. This flow cell allows generating around 400 million reads per run, therefore 45 libraries per run were loaded to guarantee around 9 million reads per sample. Calculation of qualitative scores of the NGS runs (cluster density, Passing Filter clusters, % PF, and Q-score) was done with the Real-Time Analysis software (Illumina) and checked by using the Illumina Sequencing Analysis Viewer (Illumina). In our experiments, we obtained 10,313.55 ± 142.6 Kreads/sample, with an optimal cluster density (242.67± 5.03 K/mm2), high % PF (80.38 ± 1.12) and Q30 (Q-Score) with an average value of 91.93% ± 0.58. Finally, the data were collected as FastQ files.

### Bioinformatics analysis of mature miRNAs

Reads in fastq files were processed using CLC Genomics Workbench 21.0.3 (Qiagen), a bioinformatics software that provides specific pipelines for small RNAs analysis. Adapter sequences were trimmed and sequences <15 nucleotides or without adapter nor unique molecular index were discarded. A sequential alignment strategy was used to map sequences on the reference GRCh38 human genome, using miRBase v.22.1 as annotation model (26). All the sequences recognized in miRBase were retrieved as mature miRNAs. Before normalization and DE analysis, data were filtered to include small RNAs with a minimum number of reads count (mean read count) >2. DE analysis was performed using Generalized Linear Model (GLM) and Trimmed mean of M-values (TMM) normalized counts (CPM) as input data, considering significant Benjamini-Hochberg adjusted p-values ≤ 0.05.

### Bioinformatics analysis of isomiRNAs

At first, reads in fastq files were processed by Cutadapt v2.5 (27) to trim the adapter and filter good quality reads (mean base Qphred > 30 and length range between 10 and 35 nucleotides). After quality control with FastQC (28), reads were mapped using IsoMiRmap v5 (29) both on the GRCh38 human genome assembly and the known hairpins sequences in miRBase v22. According to the IsoMiRmap method, isomiRs were identified, assigning universally unique identifiers, and quantified. Finally, the expression matrix with raw counts of exclusive-isomiRs was normalized for sequencing depth and RNA composition and DE isomiRs were assessed with DESeq2 v1.30.1 (30), considering significant Benjamini-Hochberg adjusted p-values ≤ 0.05.

### Identification of miRNA putative gene targets and network analysis

Gene targets for candidate miRNAs were identified using bioinformatics tools with online target prediction algorithm, MIENTURNET and miRWalk 3.0 (31, 32). Significant target genes for these miRNAs were selected using gene set enrichment analysis (GSEA) and implemented with Search Tool for the Retrieval Interacting Genes (STRING) v11.5, through gene ontology (GO) functional analysis and annotation databases, i.e., KEGG pathways (33), WikiPathways (34), and Disease Ontology (35), FDR<0.05. Visualization summary networks were created by Cytoscape v3.9.0 (36).

### Cells and culture conditions

Vero E6 cells (CRL-1586, American Type Culture Collection, ATCC, Manassas, VA) and Calu-3 cells (HTB-55, ATCC) were maintained in Dulbecco’s modified Eagle’s medium (DMEM, Thermo Fisher Scientific) supplemented with 20% v/v of filtered fetal bovine serum (FBS, Thermo Fisher Scientific), 1% v/v penicillin/streptomycin (Pen/Strep, Thermo Fisher Scientific) and 1% v/v of GlutaMAX supplement (Thermo Fisher Scientific). Human epithelial colorectal adenocarcinoma cell line Caco-2 (HTB-37, ATCC) was cultured in Minimum Essential Medium (Thermo Fisher Scientific) supplemented with 20% FBS, 1% Pen/Strep, and 1% GlutaMAX supplement. Primary human umbilical vein endothelial cells (HUVEC), pooled from multiple donors, were supplied by Invitrogen (Thermo Fisher Scientific) cryopreserved at the end of the primary culture stage. These cells were cultured in adhesion in Medium 200 (M200, Thermo Fisher Scientific), supplemented with 1% v/v Large Vessel Endothelial Supplement (LVES 50x, Thermo Fisher Scientific) and 1% v/v Pen/Strep. Peripheral blood mononuclear cells were purified by Ficoll-Paque PREMIUM (Merck) gradient from healthy blood donors and grown in RPMI 1640 Medium (Thermo Fisher Scientific) supplemented with 10% FBS, 1% Pen/Strep, 1% GlutaMAX and 1% Hepes. For the experiments, cells were seeded in 6-well or 12-well plates and maintained at 37°C in a humidified 5% CO2 incubator.

### SARS-CoV-2 infection and IFN-α treatment experiments.

The SARS-COV-2 isolate (lineage B1) used in infection experiments was obtained from a nasopharyngeal swab collected for diagnostic purpose. The virus was propagated in Vero E6 cells, titrated by end-point dilution assay. A lysate from uninfected Vero-E6 cells was used as a mock infection control. Infection was done at the indicated MOI for 1.5 h at 37°C to allow the adsorption of the virus. Then, the viral inoculum was removed, the cells were washed two times with PBS, and fresh medium with FBS added. Viral load was measured in cell supernatant by 50% tissue culture infective dose (TCID50) assay, as previously described (37). Viral RNA load was quantified in cells and cell supernatant by qRT-PCR. Immunofluorescence staining of SARS-CoV-2 Nucleocapsid protein was done with a rabbit monoclonal primary antibody (40143-R019; Sino Biological Inc., Beijing, China) at the dilution of 1:1000 and anti-rabbit IgG Alexa Fluor-546 secondary antibody (goat, 1:2000, Thermo Fisher Scientific). Images were achieved by Nikon Eclipse Ti confocal microscope and acquired using Nis-Element software (Nikon, Tokyo, Japan). For type I IFN stimulation experiments, cells were treated for 24 h with human recombinant IFN-α2 (Merck & Co., White House Station, NJ, USA) using a final concentration of 1,000 U/mL.

### Real-time RT-PCR analyses

Total RNA was isolated from the cells using miRNeasy Tissue/Cells advanced Mini Kit (Qiagen) and reverse transcribed to cDNA by using Murine Leukemia Virus (MuLV) reverse transcriptase (Thermo Fisher Scientific). Expression of *IFIT1*, *IFIT2*, *IL6*, *IL1B*, *TLR7*, *TLR8*, *RIG-I*, and *MDA5* mRNA was determined by real-time RT-PCR, as previously described (37). For miRNA analysis, cDNA for miRNAs was generated using TaqMan Advanced miRNA cDNA Synthesis kit (Thermo Fisher Scientific) according to manufacturer’s instructions. Then, miRNA levels were determined by real-time RT-PCR using TaqMan Fast Advanced Master mix and TaqMan Advanced miRNA Assays (Thermo Fisher Scientific) as indicated by the manufacturer. RNU6B was used as internal control for normalization of miRNA expression. Expression changes relative to mock were determined by the 2-ΔΔCT method.

### Statistical analysis

Power analysis for NGS data was computed as previously reported (38, 39). Sample size was estimated to be at least 20 subjects per group to reach the desired power of 90%, with a standard deviation (SD) estimated at 0.5, an average power with FDR of 0.05 and fold change of 2. Since our experimental design for NGS analysis provides 89 COVID-19 patients, divided into groups of more than 25 individuals, and 45 healthy controls, the sample size was considered large enough to reach the required power.

Comparisons among groups were done by Pearson’s χ^2^ test, one-way ANOVA, Student’s t test, non-parametric Wilcoxon-Mann-Whitney test, Kruskal–Wallis test, receiver operating characteristic (ROC) curve analysis, multiple logistic regression analysis, and Kaplan-Meyer survival curve analysis, as appropriate. Comparisons among multiple groups were corrected with Turkey or Bonferroni-Dunn methods. For analysis across multiple miRNAs, raw p-values were corrected for multiple testing by the Benjamini-Hochberg FDR method. Relationships between variables were assessed by Pearson correlation and Spearman’s correlation, as appropriate. Results were considered statistically significant with a P value ≤ 0.05.

### Data visualization

All the graphs and statistical analysis were done using Prism GraphPad 9.2 (Graph-Pad Software, Inc. La Jolla, CA, USA). Data in graphs and tables are reported as median and IQR, number (n) and percentage (%), mean ± standard deviation (SD), geometric mean ± SD of geometric mean. Networks of miRNA gene targets were visualized by Cytoscape v3.9.0 (36).

## Results

### Patient demographics, clinical and laboratory findings, and disease course

Overall, 89 patients with laboratory-confirmed diagnosis of acute SARS-CoV-2 infection were enrolled in the study, including 26 with mild COVID-19, 29 with moderate disease, and 34 with severe disease. Demographic and clinical features of COVID-19 patients are summarized in **Table 1**. The severity of COVID-19 was significantly associated with male sex, longer hospitalization, intensive care unit (ICU) admission, and death or long-term sequelae (**Table 1** and **Supplementary Figure 1**). The results of routine laboratory tests, which were performed at the time of hospital admission, when serum samples were also collected, are shown in **Table 1** and **Supplementary Figure 2**. The absolute number of leukocytes, the levels of CRP and the levels of creatine phosphokinase (CPK) were significantly higher in patients with severe COVID-19 than in moderate and mild COVID-19 groups.

**Table 1:** Demographics data, clinical characteristics and laboratory findings in patients with SARS-CoV-2 infection at the time of hospital admission (n = 89).

Feature	Mild COVID-19 (n = 26)	Moderate COVID-19 (n = 29)	Severe COVID-19 (n = 34)	*p* value
**Age**, years	69 (62-78)	73 (66-80)	67 (57-73)	0.1808
**Sex**				**0**.**0225**
Men	14 (53.9%)	18 (62.1%)	29 (85.3%)	
Women	12 (46.1%)	11 (37.9%)	5 (14.7%)	
**Any comorbidity**	24 (92.3%)	24 (82.8%)	29 (85.3%)	0.6871
Hypertension	13 (50.0%)	13 (44.8%)	19 (55.9%)	0.1254
Dyslipidemia	3 (11.5%)	8 (27.6%)	7 (20.6%)	0.3341
Diabetes	4 (15.4%)	8 (27.6%)	10 (29.4%)	0.3003
Malignancy	2 (7.7%)	6 (20.7%)	4 (11.8%)	0.3457
Cardiovascular Diseases	7 (26.9%)	11 (37.9%)	10 (29.4%)	0.6448
Lung disease	5 (19.2%)	8 (27.6%)	9 (26.5%)	0.739
Chronic liver disease	1 (3.9%)	1 (3.5%)	5 (14.7%)	0.169
**Days of hospitalization**	10 (8-15)	16 (10-21)	24 (13-50)	**<0**.**0001**
**ICU admission**	0 (0%)	2 (6.9%)	25 (73.5%)	**<0**.**0001**
**Outcome**				**<0**.**0001**
Recovery	23 (88.5%)	25 (86.2%)	13 (38.2%)	
Sequelae	3 (11.5%)	2 (6.9%)	11 (32.3%)	
Death	0 (0%)	2 (6.9%)	10 (29.4%)	
**Laboratory test (normal range)**				
Leukocytes count (4.3-10.8 10^9/L)	6.4 (4.8-8.5)	6.7 (5.6-9.1)	8.9 (4.9-11.0)	**0**.**0361**
CRP (<5 mg/L)	64.9 (21.2-103.3)	101.3 (36.5-171.5)	115.0 (75.0-177.2)	**0**.**0177**
Procalcitonin (<0.5 µg/L)	0.1 (0.06-0.22)	0.2 (0.09-0.46)	0.46 (0.18-1.13)	0.5418
Fibrinogen (1.8–3.5 g/L)	5.5 (5.0-6.1)	5.4 (4.4-6.2)	4.6 (3.9-6.4)	0.6501
D-dimer (<500 µg/L FEU)	820 (517.5-1095)	949 (580-1827)	857 (679-1709)	0.7089
CPK (46-171 U/L)	67.5 (43.8-139)	95 (73-186)	140 (69.8-343.3)	**0**.**0465**
Troponin (<20 ng/L)	7.4 (5.2-12.3)	11.1 (6.6-21.1)	10.1 (6.3-30)	0.5392
LDH (<247 U/L)	248 (219-311)	298.5 (255-347)	311 (273-408)	0.2913
Ferritin (23.9 – 336.2 µg/L)	424 (238-790)	408 (293-862)	697 (393-1448)	0.2042
IL-6 (<7 pg/mL)	17.1 (3.8-37.7)	42.5 (20.3-130.7)	65.3 (14.3-120.6)	0.0643

Data are median (IQR) or number (%). P values comparing mild, moderate and severe COVID-19 are from χ^2^ test and one-way ANOVA, as appropriate. CRP, C-reactive protein; CPK, creatine phosphokinase; LDH, Lactate dehydrogenase; IL-6, interleukin-6. P values of statistically significant test results defined as <0.05 are in bold.

### Association of serum miRNAs with COVID-19 and disease severity

**Figure 1:**
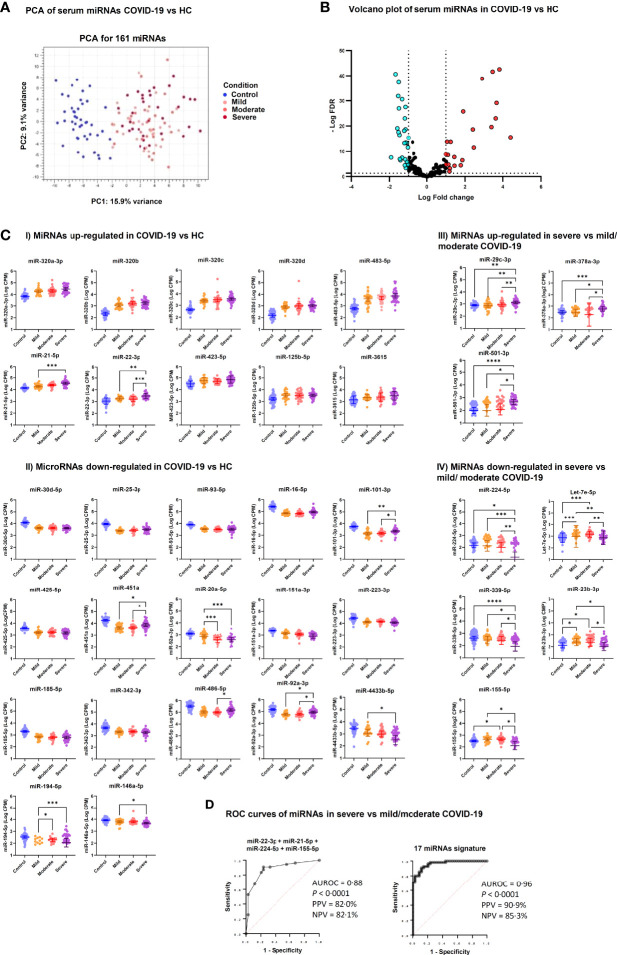
Differentially expressed serum miRNAs in COVID-19 patients. **(A)** Unsupervised principal component analysis of miRNA expression profiles in COVID-19 and HC samples (phenotype); condition is red for HC and blue, green, violet for COVID-19 severity. **(B)** Volcano plot of 161 miRNAs analyzed for differential expression (DE); vertical lines delineate > ± 2-fold change; horizontal line delineate adjusted p < 0.05. Adjusted p-values using Generalized Linear Model and trimmed mean of M-values normalized counts were used as input data and adjusted for multiple testing using the Benjamini–Hochberg false discovery rate (FDR) method; light blue dots indicate downregulated miRNA and red dots upregulated miRNAs**. (C)** Scatter dot plots of candidate DE serum miRNAs in COVID-19 patients. MicroRNA expression is reported as Log_10_ counts per million reads (Log CPM). Panels I and II show miRNAs that were up- and downregulated in severe, moderate and mild COVID-19 *vs.* HC, while Panels III and IV show DE miRNAs in severe COVID-19 *vs.* moderate and mild disease. Geometric mean values with 95% confidence interval (95% CI) are indicated by lines and error bars. Pairwise comparison between groups was done by Kruskal Wallis test for multiple comparisons, considering Benjamini-Hochberg adjusted p-values ≤ 0.05. Groups include healthy controls (Control, n = 45), mild COVID-19 (Mild, n = 26), moderate COVID-19 (Moderate, n = 29), and severe COVID-19 (Severe, n = 34). ****p<0.0001, ***p<0.001, **p<0.01, *p<0.05. Since Panels I and II include miRNAs that were DE in all COVID-19 groups (mild, moderate and severe) *vs.* HC, statistical significance is indicated only for comparisons among COVID-19 groups**. (D)** Candidate miRNA biomarkers to classify severe COVID-19 *vs.* mild/moderate COVID-19. *P* values and receiver operating characteristic (ROC) curves were calculated by multiple logistic regression analysis. AUROC, area under the ROC curve; PPV, positive predictive power; NPV, negative predictive power.

Analysis of small RNA sequencing data obtained from serum of COVID-19 patients and HC identified 161 miRNAs that were consistently expressed across groups. Principal component analysis (PCA) showed that their expression profile well discriminated between COVID-19 patients and HC (**Figure 1A**). DE analysis identified 23 upregulated miRNAs and 27 downregulated miRNAs in COVID-19 patients *vs.* HC (**Figures 1B, C** and **Supplementary Table 1**). ROC curve analysis of the DE miRNAs identified several miRNAs that could discriminate between COVID-19 patients, regardless of disease severity, and HC with high sensitivity and specificity. Among these miRNAs, high levels of miR-320 family members and miR-483-5p and low levels of miR-30d-5p, miR-25-3p, miR-93-5p, miR-16-5p showed >90% sensitivity and >90% specificity in discriminating between COVID-19 patients and HC (**Table 2**).

**Table 2:** ROC curve analysis of differentially expressed serum microRNAs in COVID-19 patients *vs.* healthy controls.

miRNA	AUC (95% CI)	*P* value	Cutpoint	Sensitivity % (95% CI)	Specificity % (95% CI)	Likelihood ratio
*Upregulated in COVID-19 vs. healthy control*						
miR-320b	0.97 (0.95-1.00)	<0.0001	> 509.3	94.4 (87.5-97.6)	95.6 (85.2-99,2)	21.24
miR-320c	0.97 (0.95-1.00)	<0.0001	> 1154	89.9 (81.9-94.6)	95.6 (85.7-99.2)	20.22
miR-320d	0.97 (0.94-0.99)	<0.0001	> 398.5	91.1 (79.3-96.5)	92.1 (84.6-96.1)	11.58
miR-483-5p	0.94 (0.91-0.98)	<0.0001	> 1371	91.0 (83.3-95.4)	91.1 (79.3-96.5)	10.24
miR-320a-3p	0.92 (0.87-0.97)	<0.0001	> 11648	80.9 (71.5-87.7)	88.9 (76.5-95.2)	7.28
miR-21-5p	0.83 (0.76-0.90)	<0.0001	> 19821	69.7 (59.5-78.2)	88.9 (76.5-95.2)	5.23
miR-22-3p	0.81 (0.74-0.89)	<0.0001	> 1478	82.0 (72.8-88.6)	66.7 (52.7-78.6)	2.46
miR-423-5p	0.79 (0.71-0.88)	<0.0001	> 38886	80.9 (71.5-87.7)	73.3 (59.0-84.0)	3.03
miR-125b-5p	0.73 (0.64-0.82)	<0.0001	> 2664	62.9 (52.6-72.3)	73.3 (59.0-84.0)	2.36
miR-3615	0.72 (0.62-0.81)	<0.0001	> 1168	88.8 (80.5-93.8)	51.1 (37.0-65.0)	1.82
*Downregulated in COVID-19 vs. healthy control*						
miR-30d-5p	0.98 (0.96-1.00)	<0.0001	< 6822	94.4 (87.5-97.6)	91,1 (79.3-96.5)	10.62
miR-25-3p	0.98 (0.95-1.00)	<0.0001	< 5160	96.6 (90.6-99.1)	91.1 (79.3-96.5)	10.87
miR-93-5p	0.97 (0.95-1.00)	<0.0001	< 5523	94.4 (87.5-97.6)	93.3 (82.1-97.7)	14.16
miR-16-5p	0.97 (0.94-1.00)	<0.0001	< 140526	92.1 (84.6-96.1)	95.6 (85.2-99.2)	20.73
miR-101-3p	0.96 (0.93-0.99)	<0.0001	< 3572	88.8 (80.5-93.8)	86.7 (73.8-93.7)	6.66
miR-185-5p	0.94 (0.90-0.98)	<0.0001	< 1094	86.8 (77.8-92.4)	88.9 (76.5-95.2)	7.81
miR-425-5p	0.92 (0.87-0.96)	<0.0001	< 2609	83.2 (74.0-89.5)	95.6 (85.2-99.2)	18.7
miR-451a	0.91 (0.87-0.96)	<0.0001	< 10205	85.4 (76.6-91.3)	82.2 (68.7-90.7)	4.80
miR-20a-5p	0.91 (0.86-0.96)	<0.0001	< 719	78.7 (69.1-85.9)	93.3 (82.1-97.7)	11.8
miR-151a-3p	0.91 (0.86-0.96)	<0.0001	< 1675	80.9 (71.5-87.7)	91.1 (79.3-96.5)	9.10
miR-223-3p	0.88 (0.80-0.95)	<0.0001	< 21788	95.5 (89.0-98.2)	71.1 (56.6-82.3)	3.31
miR-342-3p	0.85 (0.78-0.92)	<0.0001	< 3617	88.8 (80.5-93.8)	66.7 (52.1-78.6)	2.66
miR-486-5p	0.85 (0.78-0.92)	<0.0001	< 229399	85.4 (76.6-91.3)	73.3 (59.0-84.0)	3.20
miR-92a-3p	0.82 (0.75-0.90)	<0.0001	< 104438	79.8 (70.3-86.8)	68.9 (54.3-80.5)	2.56
miR-4433b-5p	0.80 (0.72-0.88)	<0.0001	< 2006	75.3 (65.4-83.1)	73.3 (59.0-84.0)	2.82
miR-194-5p	0.80 (0.72-0.87)	<0.0001	< 244	75.0 (65.0-82.9)	77.8 (63.7-87.5)	3.38
miR-146a-5p	0.75 (0.67-0.84)	<0.0001	< 7681	78.7 (69.1-85.9)	62.2 (47.6-74.9)	2.08

Furthermore, comparative analysis of serum miRNA levels among COVID-19 patients in accordance with disease severity (mild, moderate, and severe disease) identified as upregulated in patients with severe COVID-19 miR-21-5p, miR-22-3p, miR-29c-3p, miR-92a-3p, miR-101-3p, miR-194-5p, miR-378a-3p, miR-451a, miR-486-5p, miR-501-3p, and as downregulated let-7e-5p, miR-20a-5p, miR-23b-3p, miR-146a-5p, miR-155-5p, miR-224-5p, miR-339-5p and miR-4433b-5p (**Figure 1C**). Among these DE miRNAs, ROC curve analysis identified a group of miRNAs that could discriminate between severe COVID-19 and moderate/mild COVID-19 (**Table 3**). In particular, high levels of miR-22-3p and miR-21-5p showed about 90% sensitivity and >50% specificity, while low levels of miR-224-5p and miR-155-5p showed low sensitivity but >85% specificity in discriminating between severe COVID-19 and moderate/mild COVID-19. Multiple logistic regression analysis showed that a signature combining these four miRNAs improved the classification performance (area under the ROC curve, AUROC = 0.88, 95% CI 0.80-0.95, p<0.0001; negative predictive power 82.1% and positive predictive power 82.0%). Classification performance was further improved by a signature comprising the 17 DE miRNAs reported in **Table 3** (AUROC = 0.96, 95% CI 0.93-0.99, p<0.0001; negative predictive power 85.3% and positive predictive power 90.9%) (**Figure 1D**).

**Table 3:** ROC curve analysis of DE serum miRNAs in severe COVID-19 *vs.* mild and moderate COVID-19.

miRNA	AUC (95% CI)	*P* value	Cutpoint	Sensitivity % (95% CI)	Specificity % (95% CI)	Likelihood ratio
*Upregulated in severe vs. mild/moderate COVID-19*						
miR-22-3p	0.80 (0.69-0.89)	<0.0001	> 2656	89.1 (78.2-94.9)	58.8 (42.2-73.6)	2.16
miR-21-5p	0.75 (0.64-0.86)	<0.0001	> 35108	90.9 (80.4-96.1)	52.9 (36.7-68.6)	1.93
miR-101-3p	0.74 (0.64-0.85)	0.0001	> 2067	80.0 (67.6-88.5)	61.8 (45.0-76.1)	2.09
miR-194-5p	0.74 (0.64-0.85)	0.0001	> 92.01	61.1 (47.8-73.0)	82.4 (66.5-91.7)	3.46
miR-29c-3p	0.72 (0.61-0.83)	0.0005	> 1097	74.1 (61.1-83.9)	67.7 (50.8-80.9)	2.29
miR-451a	0.72 (0.60-0.83)	0.0006	> 8364	90.9 (80.4-96.1)	44.1 (28.9-60.6)	1.63
miR-92a-3p	0.71 (0.60-0.82)	0.0009	> 67074	69.1 (56.0-80.0)	70.6 (53.8-83.2)	2.35
miR-378a-3p	0.71 (0.60-0.82)	0.0012	> 609.1	75.9 (63.1-85.4)	61.8 (45.0-76.1)	1.99
miR-486-5p	0.68 (0.56-0.80)	0.0040	> 124558	72.7 (60.0-82.7)	64.7 (47.9-78.5)	2.06
miR-501-3p	0.68 (0.57-0.79)	0.0042	> 547.0	72.7 (60.0-82.7)	55.9 (39.5-71.1)	1.65
*Downregulated in severe vs. mild/moderate COVID-19*						
miR-224-5p	0.74 (0.63-0.84)	0.0002	< 255.7	60.0 (46.8-71.9)	85.3 (69.9-93.6)	4.08
let-7e-5p	0.71 (0.60-0.82)	0.0009	< 1074	72.7 (59.8-82.7)	67.7 (50.8-80.9)	2.25
miR-339-5p	0.70 (0.59-0.81)	0.0014	< 383.6	60.0 (46.8-71.9)	73.5 (56.9-85.4)	2.27
miR-23b-3p	0.67 (0.55-0.79)	0.0074	< 192.4	81.8 (69.7-90.0)	52.9 (36.7-68.6)	1.74
miR-155-5p	0.67 (0.56-0.78)	0.0075	< 509.7	43.6 (31.4-56.7)	88.2 (73.4-95.3)	3.71
miR-146a-5p	0.66 (0.55-0.78)	0.0103	< 5002	67.3 (54.1-78.2)	64.7 (47.9-78.5)	1.91

### Circulating isomiR signatures in COVID-19 patients according to disease severity

**Figure 2:**
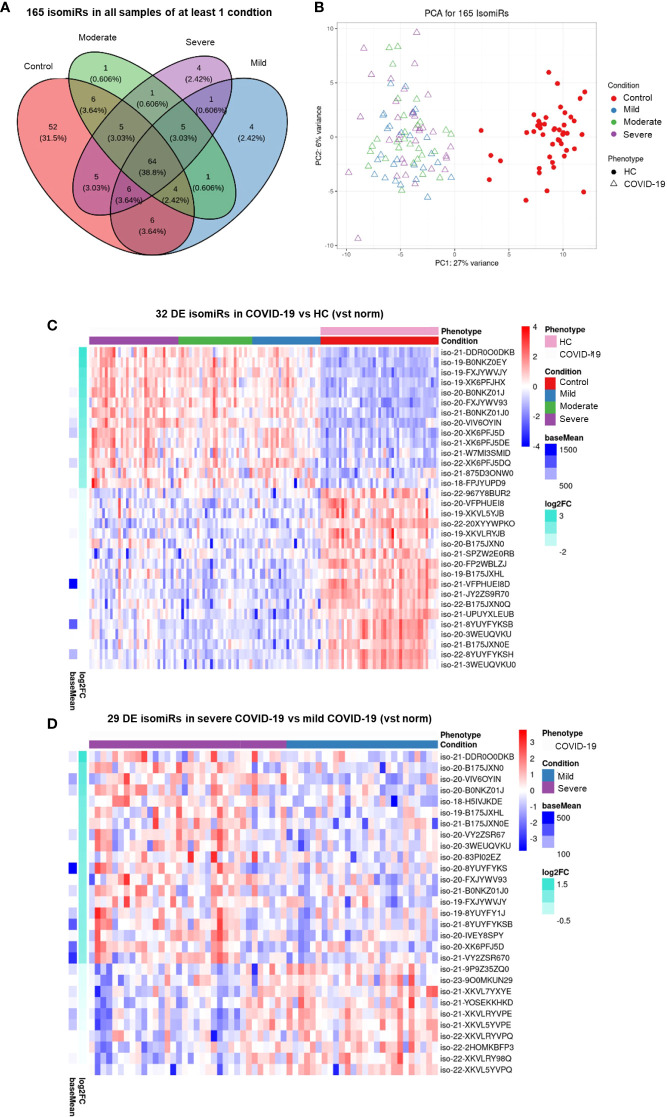
Serum isomiR analysis in COVID-19 patients. **(A)** Venn diagram showing the 165 isomiRs detected by small RNA-seq in serum samples of all individuals belonging to at least one condition. Conditions included healthy control (HC, n = 45), mild COVID-19 (n = 26), moderate COVID-19 (n = 29), and severe COVID-19 (n = 34). **(B)** Unsupervised principal component analysis of the isomiR expression profiles in COVID-19 and HC samples (phenotype); condition is red for HC and blue, green, violet for COVID-19 severity. Heatmaps **(C)** of the 32 differentially expressed (DE) serum isomiRs between COVID-19 and HC (p-value ≤ 0.01, absolute log2 Fold Change (Log2FC) ≥ 1) and **(D)** of the 29 DE serum isomiRs between severe and mild COVID-19 (p-value ≤ 0.05, absolute Log2FC ≥ 0.5); standardized expression; the baseMean column on the left indicate the mean expression for each isomiR in all samples.

-.2IsomiRs are miRNA isoforms produced during miRNA maturation that differ from their canonical counterpart in length at their 3’ or 5’ end and/or in their internal sequence. IsomiRs have variable expression in different organs and tissue types and may differ in their targeted mRNA spectrum (42). Since small RNA sequencing can identify isomiRs, we searched for DE isomiRs in our dataset. DE analysis and PCA showed that also serum isomiR levels clearly discriminated COVID-19 patients from HC, while differences among severe, moderate and mild COVID-19 conditions were less clear (**Figures 2A, B**). DE analysis between all COVID-19 patients and HC identified 122 DE serum isomiRs (58 upregulated and 64 downregulated). Among these DE isomiRs, 32 were highly expressed (above average values) and showed absolute log2 fold change >1 (**Figure 2C** and **Supplementary Table 2**). Among DE isomiRs, the top upregulated serum isomiRs in COVID-19 patients included isoforms of miR-320 family members and miR-483-5p, while the maximum downregulated serum isomiRs in COVID-19 patients included isoforms of miR-486-5p and miR-16-5p (**Supplementary Table 2**). Comparison between severe and mild COVID-19 identified 57 DE serum isomiRs, including 29 highly expressed isomiRs (above average values) with absolute log2 fold change >0.5, among which isoforms of miR-21-5p, miR-451a, and miR-22-3p as the most upregulated and isoforms of let-7 family members and miR-146a-5p among the most downregulated (**Figure 2D** and **Supplementary Table 3**).

### Signaling networks of COVID-19-associated serum miRNAs

**Figure 3:**
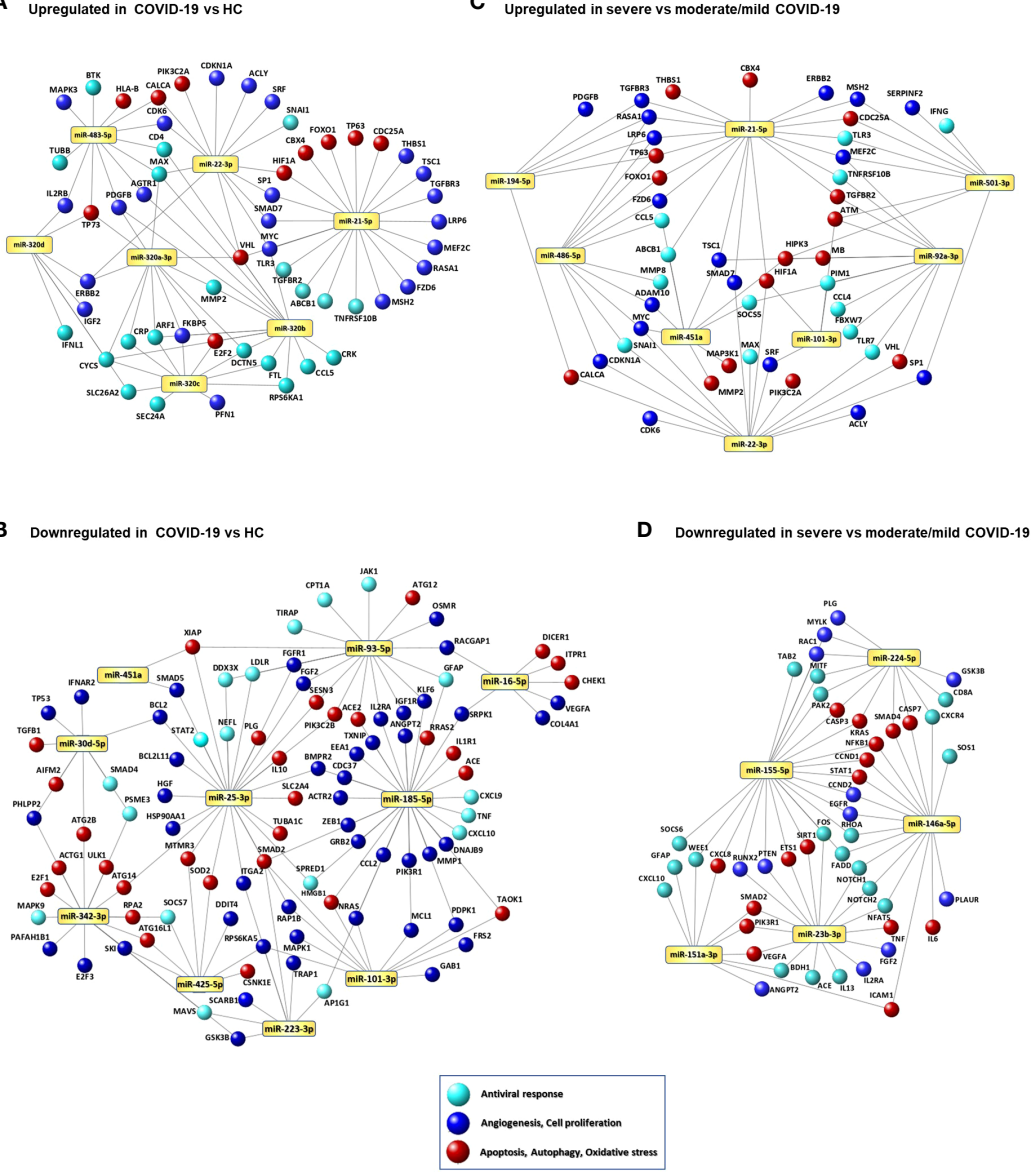
Signaling networks of COVID-19-associated serum miRNAs. Networks of putative target genes for circulating miRNAs that were significantly upregulated **(A)** and downregulated **(B)** in COVID-19 patients *vs.* healthy controls (HCs) and significantly upregulated **(C)** and downregulated **(D)** in patients with severe COVID-19 *vs.* patients with mild/moderate COVID-19. Methods for target gene identification and selection and network visualization are described in the Methods section.

Network analysis of the genes targeted by DE miRNAs in COVID-19 *vs*. HC is represented in **Figures 3A, B** and in **Supplementary Tables 4, 5**. These networks included genes involved in cell response to oxidative stress, autophagy, mitophagy, apoptosis, cell senescence, and angiogenesis. In particular, the upregulated miR-320 family targets several genes involved in antiviral defense, such as genes encoding cytokines, chemokines and cytokine receptors (*IFNL1, CCL5, IL2RB*), C-reactive protein (*CRP*), ferritin light chain (*FTL*), cytochrome c (*CYCS*), matrix metalloproteinase 2 (*MMP2*), and proteins involved in intracellular trafficking (*ARF1, DTCN5, SEC24A, SLC26A2*). Additional targets of this miRNA family include the angiotensin II receptor *AGTR1*, genes involved stress response and angiogenesis (*FKBP5, PDGFB, VHL*), and in cell proliferation (*CRK, E2F2, ERBB2, IGF2, MAX, RPS6KA11*). Targets of the upregulated miR-483-5p involved in antiviral response and angiogenesis are shared with miR-320 family gene targets. MiR-21-5p and miR-22-3p, both upregulated in COVID-19 patients, target several genes involved in cell signaling, cell proliferation and angiogenesis (e.g., *HIF1A, MYC, SP1, SMAD7, VHL*). MiRNAs that were downregulated in serum of COVID-19 patients mainly target genes promoting angiogenesis (e.g., *VEGFA, ANGPT2, COL4A1, FGF2, ZEB1*), apoptosis, autophagy, stress response (e.g., *ATG12, ATG14, ATG2B, SOD2, TXNIP*), and inflammation (e.g., *CXCL9, CXCL10, IL1R1, TNF*). The SARS-CoV-2 receptor gene ACE2 was targeted by miR-93-5p and miR-185-5p, both downregulated in COVID-19 patients.

Network analysis of the DE miRNAs in severe COVID-19 compared with mild and moderate COVID-19 are shown in **Figures 3C, D** and **Supplementary Tables 6, 7**. Upregulated miRNAs in severe COVID-19 target key antiviral response genes (*CCL4, CCL5, IFNG, STAT1, TLR3, TLR7*), genes involved in myocardial disease (*MMP2, MMP9*), lung disease (*PTEN, HMGB1, SIRT1, HIF1A, BSG, AKT1, ERBB2*), cell response to stress, autophagy, cell senescence, and angiogenesis (e.g., *IGF1R, FOXO1, PTEN, PIK3R1, CDKN1A, SIRT1, AKT1*) (**Figure 3C**), while downregulated miRNAs target genes encoding pro-inflammatory factors (*IL6, TNF, CXCL8, CXCL10*) and involved in respiratory failure, acute respiratory distress syndrome, pulmonary fibrosis, myocardial infarction, and peripheral vascular disease (*CXCR4, CXCL8, NFKB1, STAT1, ICAM1, SMAD2, IL6, RHOA, CCND1, PLAUR*) (**Figure 3D**).

### Association of laboratory parameters and serum miRNAs with COVID-19 clinical course

**Figure 4:**
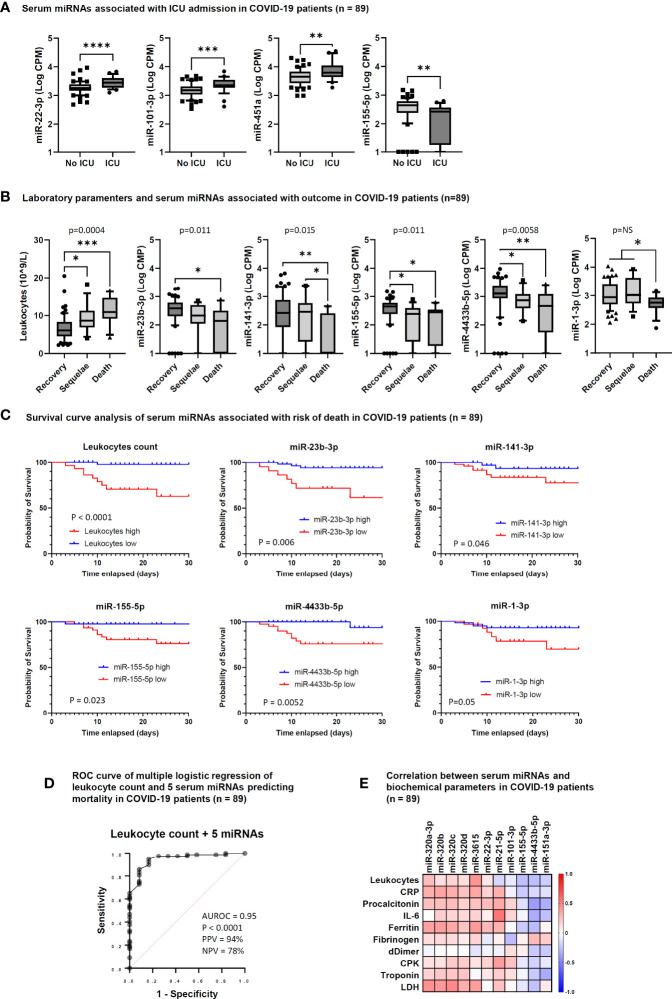
Serum microRNAs associated with intensive care unit (ICU) admission and outcome in COVID-19 patients. **(A)** Box and whiskers plot of differentially expressed (DE) miRNAs between COVID-19 patients hospitalized at ICU (ICU, n = 28) and not hospitalized at ICU (No ICU, n = 61). Wishers represent 10-90 percentile; *p* values indicated by * were determined by unpaired Mann Whitney test. **(B)** Box and whiskers plot of leukocyte counts and DE miRNAs among COVID-19 patients who recovered (Recovery, n = 61), showed COVID-19-related sequelae (Sequelae, n = 16) or had died (Death, n = 12) by day 90 after hospitalization. Wishers represent 10-90 percentile; *p* values indicated by * were determined by Kruskal Wallis test for multiple comparisons, considering two stage linear step-up procedure of Benjamini, Krieger and Yekutieli adjusted p-values ≤0.05. One-way ANOVA p value results of comparisons among the three groups are shown in the graphs. **(C)** Survival curve analysis of serum miRNAs significantly associated with the risk of death at 28 days after hospitalization. Comparisons between groups were made by Log-rank test or Gehan-Breslow-Wilcoxon test. *P* values are shown in the graphs and statistical significance was defined by p <0.05. Cut-off values for low and high miRNA levels in serum were determined by ROC curve analysis. **(D)** Candidate miRNA biomarkers to predict the risk of death in COVID-19 patients. *P* value and receiver operating characteristic (ROC) curve were calculated by multiple logistic regression analysis. AUROC, area under the ROC curve; PPV, positive predictive power; NPV, negative predictive power. The five serum miRNAs include miR-1-3p, miR-23b-3p, miR-141-3p, miR-155-5p, and miR-4433b-5p. **(E)** Correlation matrix between serum miRNAs and laboratory parameters in COVID-19 patients (n = 89). The heatmap represents Spearman *r* values of miRNAs showing one or more statistically significant correlation with any laboratory parameter. *p<0.05; **p<0.01; ***p<0.001; ****p<0.0001.

To identify biomarkers that could predict the risk of a worsened disease progression, such as ICU admission, death or development of long-term sequelae, we analyzed the results of routine laboratory tests and the levels of circulating miRNAs according to these outcome parameters. This analysis showed that COVID-19 patients admitted at ICU had significantly higher levels of serum miR-22-3p, miR-101-3p, and miR-451a, and lower levels of miR-155-5p at the time of hospitalization than patients who did not require ICU care (**Figure 4A**). At variance, no routine laboratory parameters were significantly associated with the risk of ICU admission.

Regarding COVID-19 outcome, a significant association was found with the absolute leukocyte count, since patients with long-term sequelae and those who died had increased leukocyte count (**Figure 4B**). Among miRNAs, low levels of miR-1-3p, miR-23b-3p, miR-141-3p, miR-155-5p, and miR-4433b-5p were significantly associated with COVID-19-related sequelae and/or death (**Figure 4B**).

Survival curve analysis confirmed that high leukocyte count (>9 × 10^9/L) and low serum levels of miR-1-3p, miR-23b-3p, miR-141-3p, miR-155-5p, and miR-4433b-5p at the time of hospital admission were associated with increased mortality evaluated at 28 days after hospitalization (**Figure 4C**). Multiple logistic regression analysis showed that a signature combining high leukocyte count with low levels of these five miRNAs was a good predictor of mortality (AUROC 0.95, 95% CI 0.89-1.00, p<0.0001; negative predictive power 78% and positive predictive power 94%) (**Figure 4D**).

### Correlation between circulating miRNAs and laboratory parameters in COVID-19 patients

To determine if the DE circulating miRNAs in COVID-19 patients were associated with inflammation, coagulation disorders, and myocardial damage, we performed correlation analysis between miRNAs and the laboratory parameters reported in **Table 1**. This analysis identified statistically significant positive correlations between the absolute leukocyte count and miR-3615 levels; between CRP, ferritin, LDH (inflammatory biomarkers) and the miR-320 family and miR-3615; between miR-21-5p and IL-6 and CPK (biomarkers of inflammation and myocardial damage, respectively). At variance, statistically significant negative correlations were found between miR-101-3p and fibrinogen and between miR-151a-3p and miR-4433b-3p and inflammation and myocardial damage biomarkers (procalcitonin, IL-6, and troponin) (**Figure 4E**).

Correlation analysis among the DE miRNAs in patients with severe COVID-19 displayed a strong positive association among members of the miR-320 family, miR-423-5p, and miR-3615 and between miR-16-5p and miR-451a, suggesting that these miRNAs might be co-regulated (**Supplementary Figure 3**).

### MicroRNAs associated with sex and age of COVID-19 patients

**Figure 5:**
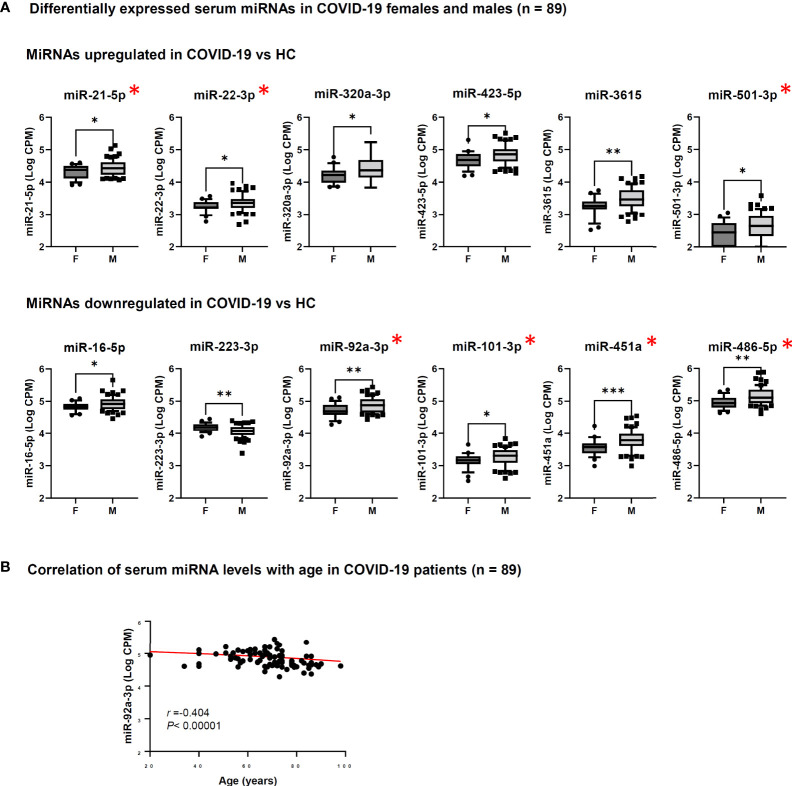
Analysis of serum miRNAs according to sex and age of COVID-19 patients. Serum miRNAs resulting significantly upregulated or downregulated in COVID-19 patients *vs.* healthy controls (HC) were included in the analyses. The red asterisk indicates miRNAs resulting significantly upregulated in patients with severe COVID-19 *vs.* mild/moderate COVID-19. **(A)** Comparison of serum miRNA levels between male (M, n = 62) and female (F, n = 27) COVID-19 patients was done by Mann-Whitney test. *p<0.05; **p<0.01; ***p<0.001. **(B)** Correlation between serum miRNA levels and age (years) of COVID-19 patients (n = 89) was done by Spearman rank correlation analysis. F, female; M, male. *r*: Spearman’s correlation coefficient. A statistically significantly correlation was found for miR-92a-3p.

Analysis of serum miRNAs according to sex of COVID-19 patients showed that the levels of several miRNAs resulting upregulated in COVID-19 *vs.* HC or in severe *vs.* mild/moderate COVID-19 (miR-21-5p, miR-22-3p, miR-92a-3p, miR-101-3p, miR-320a-3p, miR-423-5p, miR-451a, miR-486-5p, miR-501-3p, miR-3615) were significantly higher in males than in females. Moreover, serum levels of miR-223-3p, which was downregulated in COVID-19 patients *vs.* HC, were significantly lower in males than in females (**Figure 5A**). Analysis of serum miRNAs according to age found a statistically significant negative correlation between miR-92a-3p and COVID-19 patients’ age (**Figure 5B**).

### Evaluation of the DE miRNAs in *in vitro* cell models

**Figure 6:**
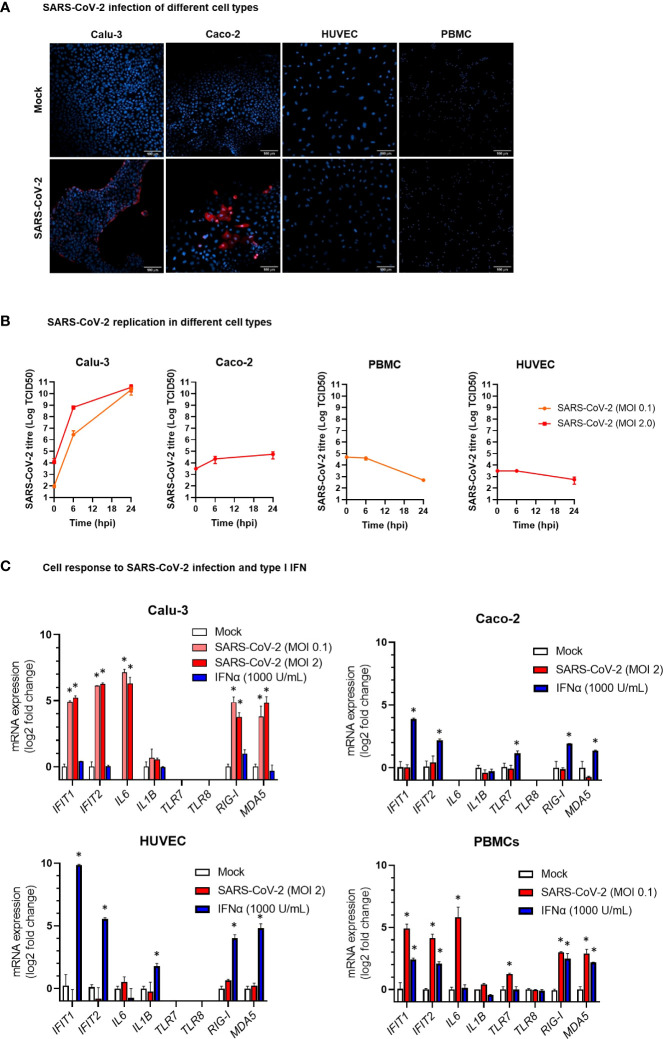
Modelling SARS-CoV-2 infection and IFNα stimulation in different human cell types. **(A)** Representative confocal microscopy images of lung carcinoma epithelial cells Calu-3, colon carcinoma epithelial cells Caco-2, human umbilical vein endothelial cells HUVEC, and peripheral blood mononuclear cells (PBMC) infected with SARS-CoV-2 or mock infected. Cells were stained with anti-SARS-CoV-2 nucleoprotein antibody (red) at 24 hours post infection. Nuclei were stained with draq5 (blue). 20× magnification. **(B)** Kinetics of SARS-CoV-2 replication in Calu-3, Caco-2, HUVEC, and PBMC. Viral load was measured by TCID50 assay in cell culture supernatant collected at different hours post infection (hpi) with SARS-CoV-2 at MOI 0.1 or 2. Viral titer is represented as mean ± SD of Log TCID50 values obtained from two experiments conducted in triplicate. **(C)** Expression of the IFN stimulated genes *IFIT1* and *IFIT2*, the pro-inflammatory cytokine genes *IL6* and *IL1B*, and the ssRNA sensor genes *TLR7* and TLR8 in HUVEC, PBMC, Caco-2 and Calu-3 cells at 24 hpi with SARS-CoV-2 at MOI 01 or 2 or treatment with IFN-α2b 1000 U/mL. mRNA expression was measured by real-time RT-PCR and represented as mean ± SD of log2 fold change *vs.* mock (calculated with the 2^-ΔΔCT^ method) obtained from two experiments conducted in triplicate. Comparison between groups (infected or treated cells *vs.* mock) was down by Mann-Whitney U test. *p<0.05.

**Figure 7:**
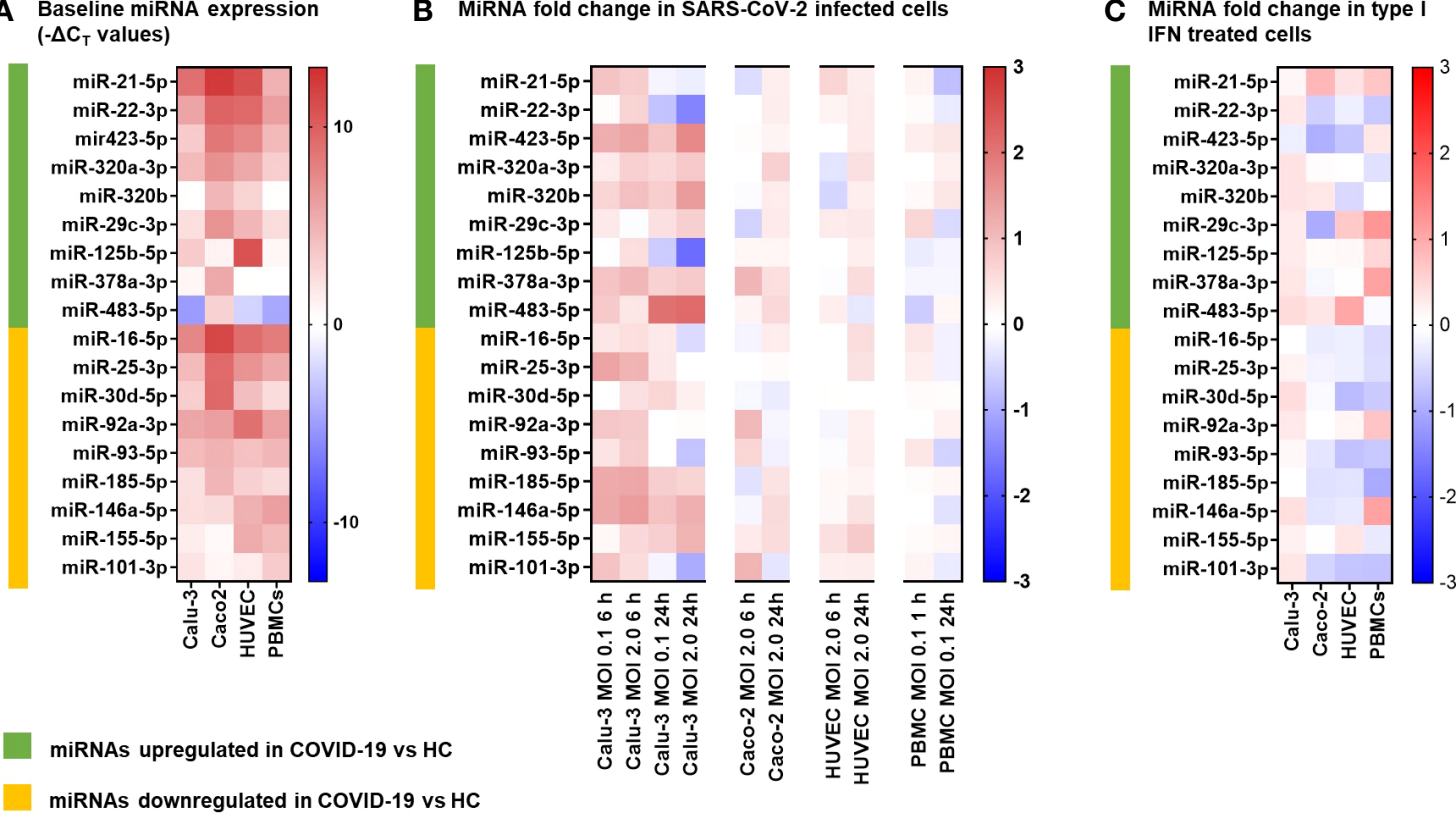
MicroRNA expression following SARS-CoV-2 infection and IFNα stimulation in different human cell types. The miRNAs investigated *in vitro* were selected among the differentially expressed (9 upregulated and 9 downregulated) serum miRNAs identified by the study in COVID-19 patients *vs.* healthy controls (HC). **(A)** Heatmap representing baseline miRNAs expression in lung carcinoma epithelial cells Calu-3, colon carcinoma epithelial cells Caco-2, human umbilical vein endothelial cells HUVEC, and peripheral blood mononuclear cells (PBMC). Data represent -ΔC_T_ values of miRNA normalized to the endogenous control *RNU6B* in triplicate samples. The color scale bar represents -ΔC_T_ values. **(B)** Heatmap representing miRNA fold change in cells infected with SARS-CoV-2 at MOI 0.1 or 2 *vs.* mock infected cells at 6 h and 24 h post infection. mRNA expression was measured by real-time RT-PCR and represented as mean log2 fold change *vs.* mock (calculated with the 2^-ΔΔCT^ method) obtained from two experiments conducted in triplicate. The color scale bar represents mean log2 fold change *vs.* mock. **(C)** Heatmap representing miRNA fold change in cells treated for 24 h with IFNα 1000 U/mL *vs.* mock treated cells. mRNA expression was measured by real-time RT-PCR and represented as mean log2 fold change *vs.* mock (calculated with the 2^-ΔΔCT^ method) obtained from two experiments conducted in triplicate. The color scale bar represents mean log2 fold change *vs.* mock.

In COVID-19 patients, circulating miRNAs could derive from cells, tissues, and organs that are directly damaged by SARS-CoV-2 infection or indirectly affected by innate immune and inflammatory responses to viral infection. To investigate the possible origin of the DE serum miRNAs, we analyzed expression of these miRNAs in human lung epithelial cancer cell line (Calu-3), human epithelial colon carcinoma cell line (Caco-2), human umbilical vein endothelial cells (HUVEC), and human PBMCs after infection with SARS-CoV-2 or treatment with type I IFN. These cells were characterized by different permissiveness to SARS-CoV-2 infection and replication (SARS-CoV-2 could efficiently infect and replicate in Calu-3 cells and, less efficiently, in Caco-2 cells, **Figures 6A, B**), responsiveness to IFN type I (Calu-3 cells did not respond to treatment with IFN-α2b, **Figure 6C**), and induction of IFN and inflammatory responses upon SARS-CoV-2 infection (induction of the viral sensors RIG-I and MDA5, the IFN response markers IFIT1 and IFIT2, and the inflammatory markers IL-6 and IL-1β in Calu-3 and in PBMCs, but not in Caco-2 and HUVEC, **Figure 6C**) and IFN type I stimulation (Calu-3 cells did not respond to IFN-α2b, **Figure 6C**). Analysis by qPCR of a subset of the DE serum miRNAs identified in COVID-19 patients showed variable expression in baseline conditions among cell types, suggesting tissue-specific expression (e.g., high levels of miR-21-5p in Caco-2 and Calu-3, miR-25-3p and miR-30d-5p in Caco-2, miR-92a-3p and miR-125b-5p in HUVEC, and miR-146a-5p in PBMCs, **Figure 7A**). Changes of miRNAs induced by SARS-CoV-2 infection were more prominent in the highly permissive Calu-3 cells than in other cell lines, with up-regulation of miR-320a-3p, miR-320b, miR-423-5p, miR-483-5p, miR-185-5p, miR-146a-5p, and miR-155-5p, while other miRNAs (miR-22-3p, miR-125b-5p, and miR-101-3p) were downregulated at 24 hpi, when cells showed cytopathic effects (**Figure 7B**). Treatment with IFN-α2b for 24 h led to upregulation of miR-21-5p in Caco-2, miR-483-5p in HUVEC, and miR-29c-3p, miR-378a-3p, and miR-146a-5p in PBMCs. At variance, treatment led to downregulation of miR-423-5p and miR-29c-3p in Caco-2 and downregulation of miR-30d-5p, miR-93-5p, miR-101-3p, miR-185-5p in HUVEC and PBMCs (**Figure 7C**).

## Discussion

**Figure 8:**
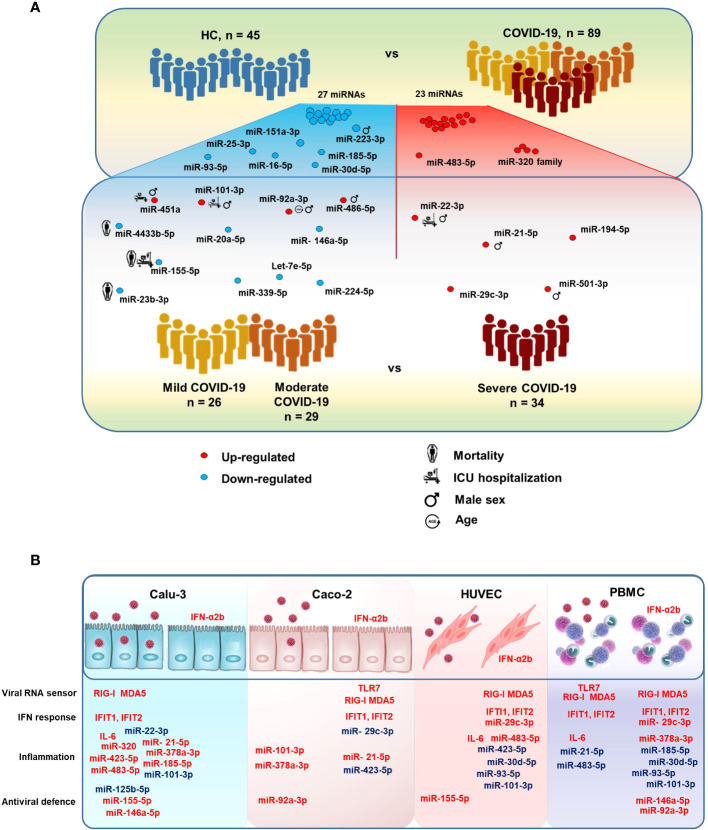
MicroRNAs modulated in COVID-19 patients. **(A)** Illustration of the study design and results, highlighting relevant serum miRNAs that were differentially expressed between COVID-19 patients *vs.* HC and between severe COVID-19 *vs.* mild and moderate COVID-19. The figure also shows serum miRNAs significantly associated with the risk of intensive care unit (ICU) hospitalization and death in COVID-19 patients, as well as male sex and age. **(B)** Illustration of the results of *in vitro* experiments, summarizing the effects of SARS-CoV-2 infection and IFN-α2b treatment on human lung (Calu-3), colon (Caco-2), endothelial (HUVEC), and peripheral blood mononuclear cells (PBMCs). Upregulated and downregulated miRNAs are represented in red and blue, respectively.

In this study, we investigated a cohort of COVID-19 patients at the time of hospital admission to discover signatures of DE circulating miRNAs associated with COVID-19 severity and disease outcome. The results of this study identified serum miRNA profiles, which could discriminate between COVID-19 and HC, between severe COVID-19 and moderate/mild disease, or predict the risk of ICU admission, COVID-19 related sequelae and death (**Figure 8A**). Expression analysis of DE miRNAs in relevant cell types *in vitro* upon SARS-CoV-2 exposure or type I IFN treatment provided hints to their possible sources (**Figure 8B**).

Through small RNA sequencing of serum miRNAs, we showed that miR-320 family members and miR-483-5p were the maximally upregulated serum miRNAs in COVID-19 patients in comparison with HC. In the literature, miR-320 family members were found as upregulated in plasma of patients with COVID-19 and especially in those with severe COVID-19 compared to those with moderate disease (17). High levels of miR-320b and miR-483-5p were also associated with increased risk of in-hospital mortality in COVID-19 patients (41) and upregulated in extracellular vesicles-enriched sera of atherosclerotic patients, indicating a possible role in vascular and endothelial injury (42). In our study, the levels of serum miR-320 family showed a positive correlation with inflammatory and tissue injury biomarkers, suggesting a role for these miRNAs in inflammatory response. Particularly strong was the positive correlation between miR-320 family and LDH, which is pathognomonic for pyroptosis and other forms of necrotic cell death and associated with severe COVID-19 (43). Interestingly, *in vitro* experiments showed that both miR-320 and miR-483-5p were significantly upregulated in lung Calu-3 cells upon SARS-CoV-2 infection.

The maximally downregulated miRNAs in serum of COVID-19 patients *vs.* HC included miR-30d-5p, miR-25-3p, miR-93-5p, miR-16-5p, miR-101-3p, miR-185-5p, miR-425-5p, miR-451a, miR-20a-5p, and miR-151-3p, which could discriminate between COVID-19 patients and HC with AUROC >0.90. These miRNAs target several pro-inflammatory cytokine and chemokine genes (e.g., *TNF, CCL2, CXCL9, CXCL10, IL10, VEGFA*) as well as cytokine and chemokine receptors and transduction factors (*IL1R1, IL2RA, IFNAR2*), reported as upregulated and associated with mortality in COVID-19 patients (44, 45). Genes involved in angiogenesis, immune cell proliferation and regulation, apoptosis, autophagy, and oxidative stress (e.g., *ACE2, ANGPT2, BCL2, FGF2, HGF, TP53*, and *ZEB1*) represented additional targets of the downregulated miRNAs in COVID-19. Since down-regulation of miRNAs leads to the up-regulation of their target genes, repression of these miRNAs in COVID-19 may contribute to the impaired innate and adaptive immune responses, the excessive systemic inflammation, the cytokine storm and cardiovascular injury that characterizes COVID-19 (46, 47).

While a miRNA signature could clearly discriminate between COVID-19 and HC, the differences in serum miRNA profile between severe COVID-19 cases and mild/moderate cases were subtle. Patients with severe COVID-19 had higher serum levels of a set of miRNAs, including miR-21-5p and miR-22-3p, which were also upregulated in COVID-19 patients *vs* HCs, in agreement with literature data (18). MiR-21-5p has been implicated in different regulatory pathways and high circulating levels were associated with lung disease and cardiac fibrosis (48, 49). Accordingly, in our COVID-19 cohort, miR-21-5p levels showed positive correlations with serum IL-6 and CPK, which are biomarkers of inflammation and myocardial damage, respectively. MiR-22-3p negatively regulates type I IFN and inflammatory cytokine production (50–52) and high circulating levels of miR-22-3p were found to predict COVID-19 mortality (17) and heart failure (53). In our study, network analysis showed that miR-21-5p and miR-22-3p share targets involved in cell signaling, cell proliferation and angiogenesis.

Additional upregulated miRNAs in severe COVID-19 compared to mild/moderate disease included miR-101-3p, miR-194-5p, miR-451a, miR-486-5p, miR-29c-3p, and miR-501-3p, while levels of circulating let-7e-5p, miR-20a-5p, miR-23b-3p, miR146a-5p, miR-155-5p, miR-224-5p, miR-339-5p, and miR-4433b-5p were significantly downregulated. Relevant targets of the up-regulated miRNAs include several genes involved in antiviral innate immune response (e.g., *IFNG, TLR3, TLR7, CCL4, CCL5*), while down-regulated miRNAs target pro-inflammatory cytokine and chemokine genes (e.g., *IL6, TNF, NFKB1, CXCL8, CXCL10, VEGFA*), which are upregulated in severe COVID-19 patients and associated with mortality (5, 45, 46). Thus, since miRNAs inhibit their target gene expression, the modulated miRNAs in severe COVID-19 would lead to the inhibition of antiviral response and to the induction of inflammation. Let-7 family is induced by IL-15 signaling in natural killer T cells, leading to IFN-γ production (54). Specifically, let-7e-5p targets *RIPK1*, *CASP8*, and *TNF*, which control signaling pathways leading to inflammation and apoptotic or necroptotic cell death. In our study, let-7e-5p levels were increased in serum of patients with mild and moderate COVID-19, but not in patients with severe disease. Likewise, other key miRNAs regulating innate antiviral response, i.e., miR-23b-3p, miR-92a-3p, miR-101-3p, miR-155-5p, miR-224-5p, miR-451a, and miR-486-5p, were modulated in patients with mild/moderate COVID-19 but not in those with severe disease. This finding is in agreement with the activation of effective antiviral responses in patients with mild and moderate COVID-19, but not in patients with severe disease, who typically have impaired innate immunity (55).

In this study, some of the circulating miRNAs that were associated with severe disease were also predictive of the clinical course and disease outcome. In particular, the levels of miR-22-3p, miR-101-3p and miR-451a were significantly higher and miR-155-5p significantly lower in patients hospitalized in ICU than in those not requiring ICU. In addition, a signature characterized by low levels of miR-1-3p, miR-23b-3p, miR-141-3p, miR-155-5p and miR-4433b-5p and high leukocyte count predicted an increased risk of COVID-19-related sequelae and/or death. Dysregulation of these miRNAs was observed is physiological and pathological processes such as immunity, inflammation, and cardiovascular diseases. In our study, circulating miR-101-3p levels showed a negative correlation with fibrinogen levels, which were low in severe COVID-19 patients and associated with poor prognosis (56). Increased serum levels of miR-101-3p were observed also in neonatal sepsis (57). MiR-451a, mostly expressed in blood cells and released in extracellular vesicles, attenuates type I IFN and IL-6 responses (58, 59) and was reported to progressively decrease with COVID-19 severity (19–21). MiR-155-5p plays a key role in the homeostasis and function of the immune system (60). It is highly expressed in activated B-cells and T-cells and in monocytes/macrophages and targets a variety of genes (61), resulting in enhancement of type I IFN signaling and subsequent innate and adaptive immune responses (62). In agreement with our data, significantly lower levels of serum miR-155-5p were found in patients with severe COVID-19 and in those who died (63). Regarding the other miRNAs associated with the risk of death and/or sequelae, miR-1-3p is muscle-specific and its expression is diminished in heart disease (64); miR-23b-3p promotes cell differentiation and inhibits cell proliferation and angiogenesis (65); miR-141-3p targets the chemokine gene *CXCL12* (66), which plays a key role in immune cell recruitment and is upregulated in severe COVID-19 (67); miR-4433b-5p is significantly down-regulated in COVID-19 patients requiring supplementary oxygen therapy (68), but its functions remain unknown. Our study showed a statistically significant negative correlation between miR-4433b-3p and inflammation and myocardial damage biomarkers suggesting it might have a role in cardiac function. This miRNA, as well as the other miRNAs predictive of mortality, warrant further research as potential therapeutic targets, once their functions in health and disease are elucidated.

Analysis of the DE serum miRNAs according to sex of COVID-19 patients, regardless of disease severity, highlighted an association between male sex and the dysregulated miRNA signature observed especially in severe COVID-19. Male patients had higher levels of serum miRNAs implicated in pro-inflammatory responses (such as miR-miR-21-5p, miR-320a-3p, miR-101-3p) and lower levels of serum miR-223-3p, which was downregulated in COVID-19 patients and identified as a negative regulator of pro-inflammatory cytokine secretion and NLRP3 inflammasome activation in the lung of SARS-CoV-2 infected mice (15). In our study, serum levels of miR-92a-3p negatively correlated with patients’ age and were down-regulated in patients with mild/moderated COVID-19 but not in those with severe disease. Interestingly, this miRNA was found to be highly expressed in mesenchymal stem-cell-derived extracellular vesicles and to target both a conserved 3’-untranslated region of SARS-CoV-2 genome and inflammatory response genes (69).

Taken together, our results from the clinical study showed that COVID-19 patients had a circulating miRNA signature characterized by upregulation of miRNAs associated with lung disease, vascular damage and inflammation and downregulation of miRNAs that inhibit expression and activity of pro-inflammatory cytokines and chemokines, angiogenesis, and stress response. Compared to patients with mild/moderate COVID-19, patients with severe COVID-19 and hospitalized in ICU had a circulating miRNA signature indicating a profound impairment of innate and adaptive immune responses, inflammation, cytokine storm, lung fibrosis and heart failure. A subset of the DE miRNAs predicted mortality in COVID-19 patients.

Circulating miRNAs are released by various cell types, mostly macrophages, lymphocytes, endothelial cells, and platelets, but also by passive leakage from damaged cells as a consequence of tissue injury, inflammation, necrosis, or apoptosis (70).

To search for the possible source of the DE circulating miRNAs in COVID-19 patients, we analyzed the expression of these miRNAs *in vitro* in relevant cell types, i.e., lung carcinoma epithelial cells Calu-3, colon carcinoma epithelial cells Caco-2, endothelial cells HUVEC, and PBMCs, characterized by different tissue origin, permissiveness to SARS-CoV-2 infection and replication, capacity to sense RNA viruses and trigger antiviral response, and integrity of type I IFN response pathway. These cells are representative of the main tissues involved in COVID-19 pathogenesis, i.e., the pulmonary epithelium, which is the primary target of SARS-CoV-2 infection and injury, and the gut epithelium, which is also productively infected by SARS-CoV-2 *in vivo* (71). Endothelial cells express low levels of ACE2 and TMPRSS2 (72) and are poorly susceptible to SARS-CoV-2 infection (71). However, endothelial cells are involved in COVID-19 pathogenesis with endothelitis and thrombo-embolic manifestations and, in animal models, SARS-CoV-2 spike protein or its S1 subunit can cause endothelial damage (73, 74). Blood cells are not productively infected by SARS-CoV-2 but can sense the virus activating innate antiviral responses (55). Accordingly, our experiments showed that Calu-3 cells were permissive to SARS-CoV-2 replication, which induced CPE and triggered IFN and inflammatory responses. The virus replicated less efficiently in Caco-2 cells without CPE nor induction of IFN or inflammatory response. PBMC stimulation with SARS-CoV-2 induced the expression of IFN stimulated genes (ISGs), while HUVEC were not infected by SARS-CoV-2 nor responded to the virus.

The results of miRNA analysis in these *in vitro* models highlighted cell-specific differences of miRNA levels in baseline conditions. Upon SARS-CoV-2 infection, most changes in intracellular miRNA levels occurred in the highly permissive epithelial lung carcinoma cell line Calu-3. In these cells, SARS-CoV-2 infection upregulated miR-185-5p, miR-320, miR-423-5p, and miR-483-5p, which promote inflammation and inhibit antiviral responses, as well as miR-146a-5p and miR-155-5p, which act antagonistically to produce a robust inflammatory response (11). Upregulation of miR-155-5p following SARS-CoV-2 infection was previously described in Calu-3 cells and associated with induction of antiviral and pro-inflammatory responses triggered by sensors of RNA viruses (75). Conversely, SARS-CoV-2 infection of Calu-3 cells led to down-regulation of miR-22-3p and miR-125b-5p. These miRNAs play a key role in the regulation of cell self-renewal, differentiation, autophagy and their overexpression lead to uncontrolled cell proliferation and defective differentiation *via* TGFβ and Wnt signaling pathways and DNA methylation (11). This miRNA signature was in agreement with the robust activation of the ISGs *IFIT1* and *IFIT2* and the pro-inflammatory cytokine genes *IL6* and *IL1B* and upon SARS-CoV-2 infection, which was conceivably sensed by RIG-I and MDA5. The colon carcinoma cell line Caco-2, which is permissive to SARS-CoV-2 but unable to activate innate antiviral and IFN responses, and the non-permissive HUVEC and PBMCs did not show relevant changes of miRNA expression upon SARS-CoV-2 infection. However, theses miRNA responses may not be totally representative of miRNA expression profiles in healthy primary lung cell or gut cells experiencing SARS-CoV-2, since the colorectal and lung cells used in this study were derived from cancer cell lines. Likewise, the umbilical endothelial cells might not be representative of the endothelial cells in the lung vasculature and PBMCs may not fully represent local immune-inflammatory cells in the alveoli.

Treatment with IFN-α modulated expression of some of the selected DE miRNAs in the IFN-responsive Caco-2, HUVEC and PBMCs, but not in Calu-3 cells, characterized by a poor response to type I IFN stimulation. In Caco-2, HUVEC, and PBMC, several miRNAs downregulated in COVID-19, such as miR-93-5p, miR-185-5p, and miR-101-3p, were consistently downregulated by IFNα stimulation. In PBMC, IFNα treatment upregulated miR-29c-3p (upregulated in serum of COVID-19 patients), which is known to target the IFN receptor *IFNAR1* as negative feedback to limit type I IFN response (76), as well as miR-146a-5p and miR-378a-3p, which, respectively, inhibit TLR-mediated innate immune responses (11) and promote NET formation by granulocytes in sepsis (77). Another overexpressed miRNA in the serum of COVID-19 patients with severe disease, miR-483-5p, was markedly induced by IFN-α treatment of HUVEC endothelial cells and by SARS-CoV-2 infection in Calu-3 cells. Overexpression of this miRNA, which leads to suppression of cell proliferation and production of inflammatory cytokines (78) was observed in the lung tissues of mice with sepsis-induced acute lung injury (79).

In conclusion, this study discovered signatures of circulating miRNAs associated with COVID-19 severity and mortality, which warrant further investigation and validation as candidate prognostic biomarkers. The identified DE circulating miRNAs provided clues on COVID-19 pathogenesis, highlighting signatures of impaired IFN and antiviral responses, inflammation, organ damage and cardiovascular failure as associated with severe disease and death. *In vitro* experiments showed that some of these miRNAs were modulated directly by SARS-CoV-2 infection or indirectly by IFN.

## Data availability statement

The datasets presented in this study can be found in online repositories. The names of the repository/repositories and accession number(s) can be found below: Gene Expression Omnibus under accession number GSE201790.

## Ethics statement

The studies involving human participants were reviewed and approved by Comitato Etico per la Sperimentazione Clinica delle Province di Verona e Rovigo. The patients/participants provided their written informed consent to participate in this study.

## Author contributions

AG, SR, AS, CP, RM, FG, GC, and LB contributed to the study design. CP, ER, and FG contributed to patient recruitment, data collection, and clinical analysis. AG, SR, AS, MA, and EM contributed to wet-lab data generation and *in vitro* analysis. AG, PB, GC, and LB contributed to small RNA sequencing analysis and statistical analysis. AG, GC, and LB wrote and revised the manuscript. All authors discussed and commented on the manuscript. RM, FG, GC, and LB provided funding to support this study. All authors contributed to the article and approved the submitted version.

## Funding

This work was funded by the European Union’s Horizon 2020 Research and Innovation Program, under grant agreement no. 874735 (VEO), and by PRID grant from the University of Padova. The work at IRCCS Sacro Cuore Don Calabria Hospital was supported by the Italian Ministry of Health “Fondi Ricerca Corrente – L1P6” and by the Italian Ministry of Health - COVID-2020-12371675. Small RNA-sequencing was performed by a NextSeq 550 Instrument purchased by the DIMAR Excellence project funding (DImed and MAlattie Rare) of the Department of Medicine, University of Padua.

## Acknowledgments

We wish to thank Andrea Benetti (Department of Medicine, University of Padova) for technical assistance.

## Conflict of interest

The authors declare that the research was conducted in the absence of any commercial or financial relationships that could be construed as a potential conflict of interest.

## Publisher’s note

All claims expressed in this article are solely those of the authors and do not necessarily represent those of their affiliated organizations, or those of the publisher, the editors and the reviewers. Any product that may be evaluated in this article, or claim that may be made by its manufacturer, is not guaranteed or endorsed by the publisher.
