# Supplementary material for: Circulating microRNA signatures associated with disease severity and outcome in COVID-19 patients
Source: Front Immunol. 2022 Aug 11;13:968991. doi: 10.3389/fimmu.2022.968991 (PMC9403711; doi:10.3389/fimmu.2022.968991)
Supplement: Supplementary file 1 [file DataSheet_1.pdf]

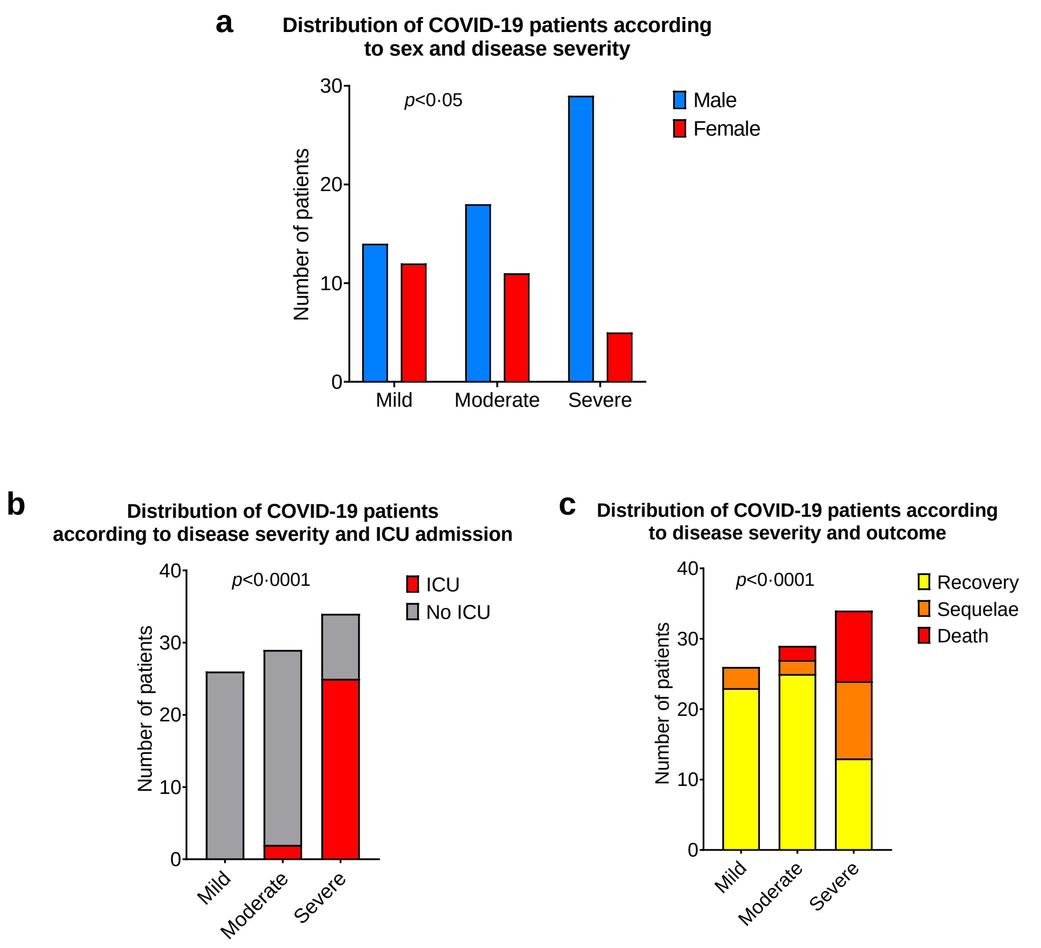

Supplementary Figure I. Distribution of COVID-19 patients (n = 89) according to disease severity and **(a)** sex, **(b)** admission to intensive care unit (ICU) and **(c)** disease outcome. Comparisons among groups were done by χ^2 test. *P* values are shown in the figure.
