# Supplementary material for: Circulating microRNA signatures associated with disease severity and outcome in COVID-19 patients
Source: Front Immunol. 2022 Aug 11;13:968991. doi: 10.3389/fimmu.2022.968991 (PMC9403711; doi:10.3389/fimmu.2022.968991)
Supplement: Supplementary file 2 [file DataSheet_2.pdf]

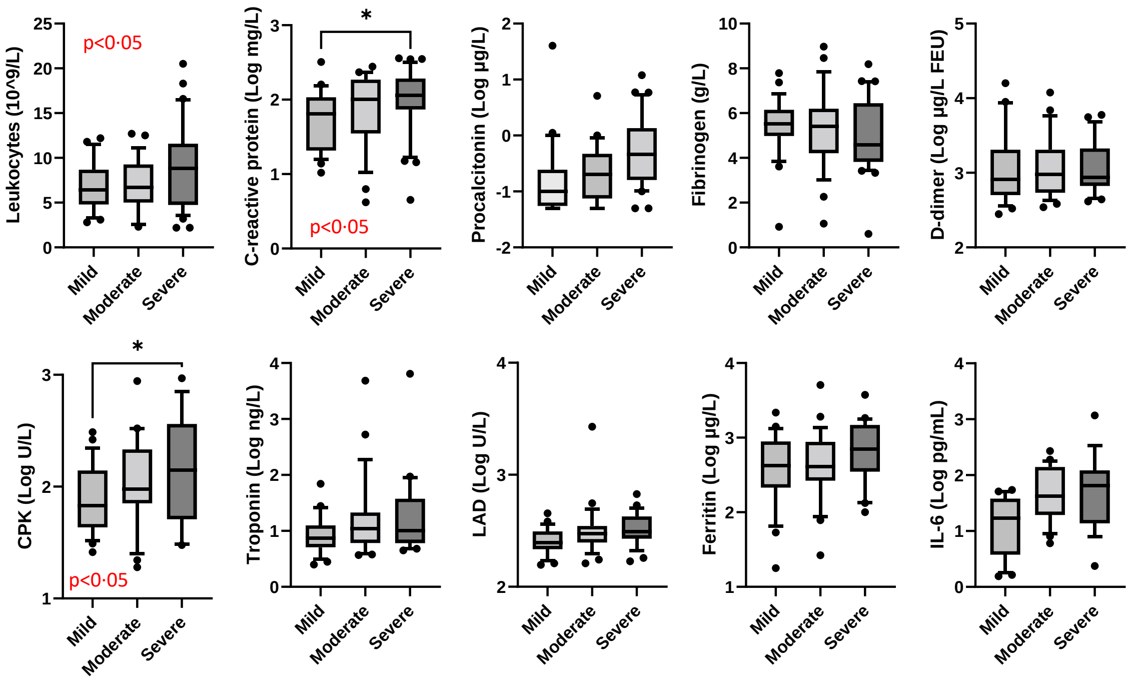

Supplementary Figure 2. Laboratory findings in COVID-19 patients at the time of hospital admission (n = 89). Data are represented as box whiskers plot, with wiskers showing 10-90 percentiles. Comparison among groups was done by one-way ANOVA with Turkey's correction for multiple analyses. Statisticaly significant p values (*p<0·05) by ANOVA are shown in the figure.
