# Supplementary material for: Circulating microRNA signatures associated with disease severity and outcome in COVID-19 patients
Source: Front Immunol. 2022 Aug 11;13:968991. doi: 10.3389/fimmu.2022.968991 (PMC9403711; doi:10.3389/fimmu.2022.968991)
Supplement: Supplementary file 3 [file DataSheet_3.pdf]

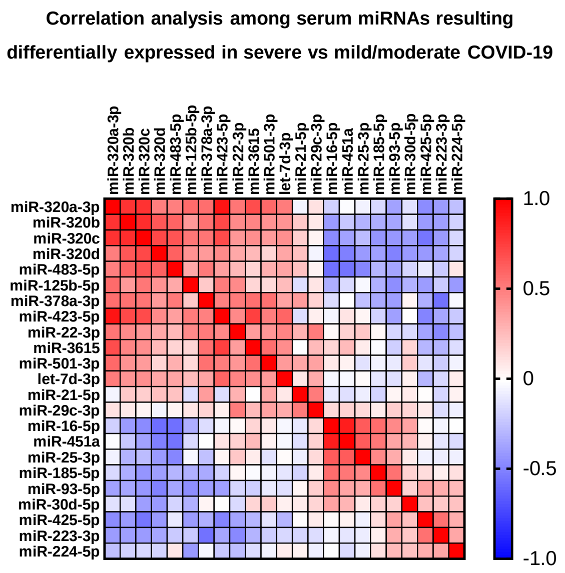

Supplementary Figure 3. Correlation matrix of differentially expressed serum miRNAs in patients with severe COVID-19 (n = 34). Analysis was done by Spearman rank correlation analysis. The heatmap represent Spearman r values of miRNAs resulting statistically significant in at least one comparison.
