# Supplementary material for: Circulating microRNA signatures associated with disease severity and outcome in COVID-19 patients
Source: Front Immunol. 2022 Aug 11;13:968991. doi: 10.3389/fimmu.2022.968991 (PMC9403711; doi:10.3389/fimmu.2022.968991)
Supplement: Supplementary file 4 [file Table_1.docx]

**Supplementary Table 1.** Differentially expressed microRNAs in COVID-19 patients (n = 89) vs. healthy controls (HC, n = 45).

| **Name** | **Identifier** | **COVID-19 vs. HC - Log fold change** | **COVID-19 vs. HC - FDR p-value** |
| --- | --- | --- | --- |
| hsa-miR-483-5p | [MI0002467](https://www.mirbase.org/cgi-bin/mirna_entry.pl?acc=MI0002467) | 3,826877 | 2,61174E-43 |
| hsa-miR-320c | [MI0003778](https://www.mirbase.org/cgi-bin/mirna_entry.pl?acc=MI0003778) | 3,468043 | 2,30527E-42 |
| hsa-miR-16-5p | [MI0000070](https://www.mirbase.org/cgi-bin/mirna_entry.pl?acc=MI0000070) | -1,679553 | 2,1738E-41 |
| hsa-miR-320b | [MI0003776](https://www.mirbase.org/cgi-bin/mirna_entry.pl?acc=MI0003776) | 2,91204 | 1,52467E-39 |
| hsa-miR-25-3p | [MI0000082](https://www.mirbase.org/cgi-bin/mirna_entry.pl?acc=MI0000082) | -1,513104 | 4,28757E-38 |
| hsa-miR-101-3p | [MI0000103](https://www.mirbase.org/cgi-bin/mirna_entry.pl?acc=MI0000103) | -1,544114 | 1,41504E-32 |
| hsa-miR-30d-5p | [MI0000255](https://www.mirbase.org/cgi-bin/mirna_entry.pl?acc=MI0000255) | -1,337248 | 2,41712E-31 |
| hsa-miR-320d | [MI0008190](https://www.mirbase.org/cgi-bin/mirna_entry.pl?acc=MI0008190) | 3,675754 | 7,22082E-30 |
| hsa-miR-93-5p | [MI0000095](https://www.mirbase.org/cgi-bin/mirna_entry.pl?acc=MI0000095) | -1,15986 | 3,1062E-28 |
| hsa-miR-185-5p | [MI0000482](https://www.mirbase.org/cgi-bin/mirna_entry.pl?acc=MI0000482) | -1,504419 | 1,06065E-27 |
| hsa-miR-320a-3p | [MI0000542](https://www.mirbase.org/cgi-bin/mirna_entry.pl?acc=MI0000542) | 1,910111 | 1,63189E-26 |
| hsa-miR-1290 | [MI0006352](https://www.mirbase.org/cgi-bin/mirna_entry.pl?acc=MI0006352) | 3,630301 | 9,59E-24 |
| hsa-miR-223-3p | [MI0000300](https://www.mirbase.org/cgi-bin/mirna_entry.pl?acc=MI0000300) | -1,191008 | 9,90328E-24 |
| hsa-miR-4516 | [MI0016882](https://www.mirbase.org/cgi-bin/mirna_entry.pl?acc=MI0016882) | 3,407438 | 2,51816E-20 |
| hsa-miR-451a | [MI0001729](https://www.mirbase.org/cgi-bin/mirna_entry.pl?acc=MI0001729) | -1,584483 | 8,16765E-20 |
| hsa-miR-425-5p | [MI0001448](https://www.mirbase.org/cgi-bin/mirna_entry.pl?acc=MI0001448) | -1,158219 | 2,04351E-19 |
| hsa-miR-760 | [MI0005567](https://www.mirbase.org/cgi-bin/mirna_entry.pl?acc=MI0005567) | 2,41301 | 2,24563E-19 |
| hsa-miR-342-3p | [MI0000805](https://www.mirbase.org/cgi-bin/mirna_entry.pl?acc=MI0000805) | -1,223563 | 6,8529E-19 |
| hsa-miR-186-5p | [MI0000483](https://www.mirbase.org/cgi-bin/mirna_entry.pl?acc=MI0000483) | -1,525454 | 2,62193E-18 |
| hsa-miR-486-5p | [MI0002470](https://www.mirbase.org/cgi-bin/mirna_entry.pl?acc=MI0002470) | -1,472851 | 3,69823E-17 |
| hsa-miR-203a-3p | [MI0000283](https://www.mirbase.org/cgi-bin/mirna_entry.pl?acc=MI0000283) | 4,407374 | 3,01927E-16 |
| hsa-miR-151a-3p | [MI0000809](https://www.mirbase.org/cgi-bin/mirna_entry.pl?acc=MI0000809) | -0,99588 | 3,95661E-16 |
| hsa-miR-21-5p | [MI0000077](https://www.mirbase.org/cgi-bin/mirna_entry.pl?acc=MI0000077) | 1,062573 | 1,42032E-14 |
| hsa-miR-423-5p | [MI0001445](https://www.mirbase.org/cgi-bin/mirna_entry.pl?acc=MI0001445) | 1,252046 | 1,61545E-14 |
| hsa-miR-92a-3p | [MI0000093](https://www.mirbase.org/cgi-bin/mirna_entry.pl?acc=MI0000093) | -1,139062 | 3,0172E-14 |
| hsa-miR-181a-5p | [MI0000269](https://www.mirbase.org/cgi-bin/mirna_entry.pl?acc=MI0000269) | -1,087367 | 4,36553E-14 |
| hsa-miR-20a-5p | [MI0000076](https://www.mirbase.org/cgi-bin/mirna_entry.pl?acc=MI0000076) | -1,2137 | 5,02628E-14 |
| hsa-miR-148b-3p | [MI0000811](https://www.mirbase.org/cgi-bin/mirna_entry.pl?acc=MI0000811) | -1,031002 | 2,55684E-12 |
| hsa-miR-1246 | [MI0006381](https://www.mirbase.org/cgi-bin/mirna_entry.pl?acc=MI0006381) | 2,46504 | 2,79501E-12 |
| hsa-miR-22-3p | [MI0000078](https://www.mirbase.org/cgi-bin/mirna_entry.pl?acc=MI0000078) | 0,99635 | 1,40899E-09 |
| hsa-let-7e-5p | [MI0000066](https://www.mirbase.org/cgi-bin/mirna_entry.pl?acc=MI0000066) | 1,106353 | 2,52195E-09 |
| hsa-miR-501-3p | [MI0003185](https://www.mirbase.org/cgi-bin/mirna_entry.pl?acc=MI0003185) | 1,438952 | 1,73091E-08 |
| hsa-miR-324-5p | [MI0000813](https://www.mirbase.org/cgi-bin/mirna_entry.pl?acc=MI0000813) | -1,913568 | 2,44985E-08 |
| hsa-miR-4433b-5p | [MI0025511](https://www.mirbase.org/cgi-bin/mirna_entry.pl?acc=MI0025511) | -1,337737 | 3,18154E-08 |
| hsa-miR-199a-3p | [MI0000242](https://www.mirbase.org/cgi-bin/mirna_entry.pl?acc=MI0000242) | -0,610023 | 3,18154E-08 |
| hsa-miR-1306-5p | [MI0006443](https://www.mirbase.org/cgi-bin/mirna_entry.pl?acc=MI0006443) | -0,945393 | 3,56394E-08 |
| hsa-miR-30e-5p | [MI0000749](https://www.mirbase.org/cgi-bin/mirna_entry.pl?acc=MI0000749) | -0,704679 | 1,03355E-07 |
| hsa-miR-4732-3p | [MI0017369](https://www.mirbase.org/cgi-bin/mirna_entry.pl?acc=MI0017369) | -1,452646 | 1,1768E-07 |
| hsa-miR-432-5p | [MI0003133](https://www.mirbase.org/cgi-bin/mirna_entry.pl?acc=MI0003133) | -1,176053 | 1,54592E-07 |
| hsa-miR-629-5p | [MI0003643](https://www.mirbase.org/cgi-bin/mirna_entry.pl?acc=MI0003643) | 0,896072 | 2,00123E-07 |
| hsa-miR-222-3p | [MI0000299](https://www.mirbase.org/cgi-bin/mirna_entry.pl?acc=MI0000299) | 0,902515 | 2,38843E-07 |
| hsa-miR-141-3p | [MI0000457](https://www.mirbase.org/cgi-bin/mirna_entry.pl?acc=MI0000457) | 1,862557 | 3,19864E-07 |
| hsa-miR-196b-5p | [MI0001150](https://www.mirbase.org/cgi-bin/mirna_entry.pl?acc=MI0001150) | -1,426303 | 3,53235E-07 |
| hsa-miR-223-5p | [MI0000300](https://www.mirbase.org/cgi-bin/mirna_entry.pl?acc=MI0000300) | -0,783001 | 5,58292E-07 |
| hsa-miR-125b-5p | [MI0000446](https://www.mirbase.org/cgi-bin/mirna_entry.pl?acc=MI0000446) | 1,013452 | 1,02739E-06 |
| hsa-miR-20b-5p | [MI0001519](https://www.mirbase.org/cgi-bin/mirna_entry.pl?acc=MI0001519) | -0,962571 | 1,02739E-06 |
| hsa-miR-340-5p | [MI0000802](https://www.mirbase.org/cgi-bin/mirna_entry.pl?acc=MI0000802) | -1,082793 | 1,33711E-06 |
| hsa-miR-106b-3p | [MI0000734](https://www.mirbase.org/cgi-bin/mirna_entry.pl?acc=MI0000734) | -0,849074 | 5,00469E-06 |
| hsa-let-7c-5p | [MI0000064](https://www.mirbase.org/cgi-bin/mirna_entry.pl?acc=MI0000064) | 1,134428 | 1,62658E-05 |
| hsa-miR-382-5p | [MI0000790](https://www.mirbase.org/cgi-bin/mirna_entry.pl?acc=MI0000790) | -1,035911 | 1,78844E-05 |
| hsa-miR-3615 | [MI0016005](https://www.mirbase.org/cgi-bin/mirna_entry.pl?acc=MI0016005) | 0,995144 | 1,78844E-05 |
| hsa-miR-103a-3p | [MI0000108](https://www.mirbase.org/cgi-bin/mirna_entry.pl?acc=MI0000108) | 0,489632 | 1,78844E-05 |
| hsa-miR-205-5p | [MI0000285](https://www.mirbase.org/cgi-bin/mirna_entry.pl?acc=MI0000285) | 1,778475 | 3,56286E-05 |
| hsa-miR-431-5p | [MI0001721](https://www.mirbase.org/cgi-bin/mirna_entry.pl?acc=MI0001721) | -1,192869 | 3,566E-05 |
| hsa-miR-378a-3p | [MI0000786](https://www.mirbase.org/cgi-bin/mirna_entry.pl?acc=MI0000786) | 0,877119 | 5,16738E-05 |
| hsa-miR-3168 | [MI0014199](https://www.mirbase.org/cgi-bin/mirna_entry.pl?acc=MI0014199) | 1,450463 | 5,26035E-05 |
| hsa-miR-19b-3p | [MI0000074](https://www.mirbase.org/cgi-bin/mirna_entry.pl?acc=MI0000074) | -0,765664 | 6,15695E-05 |
| hsa-miR-409-3p | [MI0001735](https://www.mirbase.org/cgi-bin/mirna_entry.pl?acc=MI0001735) | -0,974629 | 6,19068E-05 |
| hsa-miR-23b-3p | [MI0000439](https://www.mirbase.org/cgi-bin/mirna_entry.pl?acc=MI0000439) | 0,853206 | 6,66326E-05 |
| hsa-miR-197-3p | [MI0000239](https://www.mirbase.org/cgi-bin/mirna_entry.pl?acc=MI0000239) | -0,716934 | 8,22806E-05 |
| hsa-miR-361-5p | [MI0000760](https://www.mirbase.org/cgi-bin/mirna_entry.pl?acc=MI0000760) | 0,726655 | 8,73708E-05 |
| hsa-miR-142-3p | [MI0000458](https://www.mirbase.org/cgi-bin/mirna_entry.pl?acc=MI0000458) | 0,487687 | 0,000104612 |
| hsa-miR-32-5p | [MI0000090](https://www.mirbase.org/cgi-bin/mirna_entry.pl?acc=MI0000090) | 0,754042 | 0,000104612 |
| hsa-miR-150-5p | [MI0000479](https://www.mirbase.org/cgi-bin/mirna_entry.pl?acc=MI0000479) | -0,607362 | 0,000135988 |
| hsa-miR-29c-3p | [MI0000735](https://www.mirbase.org/cgi-bin/mirna_entry.pl?acc=MI0000735) | 0,687698 | 0,000143761 |
| hsa-miR-193a-5p | [MI0000487](https://www.mirbase.org/cgi-bin/mirna_entry.pl?acc=MI0000487) | 0,930471 | 0,000175083 |
| hsa-let-7d-3p | [MI0000065](https://www.mirbase.org/cgi-bin/mirna_entry.pl?acc=MI0000065) | 0,556876 | 0,000346094 |
| hsa-miR-485-3p | [MI0002469](https://www.mirbase.org/cgi-bin/mirna_entry.pl?acc=MI0002469) | -1,142841 | 0,000415543 |
| hsa-miR-19a-3p | [MI0000073](https://www.mirbase.org/cgi-bin/mirna_entry.pl?acc=MI0000073) | -0,926203 | 0,000429534 |
| hsa-miR-1-3p | [MI0000437](https://www.mirbase.org/cgi-bin/mirna_entry.pl?acc=MI0000437) | 0,996547 | 0,000555854 |
| hsa-miR-4508 | [MI0016872](https://www.mirbase.org/cgi-bin/mirna_entry.pl?acc=MI0016872) | 1,208864 | 0,000570765 |
| hsa-miR-126-3p | [MI0000471](https://www.mirbase.org/cgi-bin/mirna_entry.pl?acc=MI0000471) | 0,356357 | 0,000575229 |
| hsa-miR-28-3p | [MI0000086](https://www.mirbase.org/cgi-bin/mirna_entry.pl?acc=MI0000086) | -0,439873 | 0,000695174 |
| hsa-miR-146a-5p | [MI0000477](https://www.mirbase.org/cgi-bin/mirna_entry.pl?acc=MI0000477) | -0,995376 | 0,000971454 |
| hsa-miR-146a-5p | [MI0000477](https://www.mirbase.org/cgi-bin/mirna_entry.pl?acc=MI0000477) | -0,464376 | 0,000971454 |
| hsa-miR-326 | [MI0000808](https://www.mirbase.org/cgi-bin/mirna_entry.pl?acc=MI0000808) | -0,87258 | 0,000971809 |
| hsa-miR-3613-5p | [MI0016003](https://www.mirbase.org/cgi-bin/mirna_entry.pl?acc=MI0016003) | -0,797811 | 0,000971809 |
| hsa-miR-363-3p | [MI0000764](https://www.mirbase.org/cgi-bin/mirna_entry.pl?acc=MI0000764) | -0,621911 | 0,001292224 |
| hsa-miR-454-3p | [MI0003820](https://www.mirbase.org/cgi-bin/mirna_entry.pl?acc=MI0003820) | 0,625612 | 0,001292224 |
| hsa-miR-142-5p | [MI0000458](https://www.mirbase.org/cgi-bin/mirna_entry.pl?acc=MI0000458) | -0,447881 | 0,00143152 |
| hsa-let-7g-5p | [MI0000433](https://www.mirbase.org/cgi-bin/mirna_entry.pl?acc=MI0000433) | -0,367028 | 0,001545327 |
| hsa-miR-139-3p | [MI0000261](https://www.mirbase.org/cgi-bin/mirna_entry.pl?acc=MI0000261) | 0,632355 | 0,003212815 |
| hsa-miR-17-5p | [MI0000071](https://www.mirbase.org/cgi-bin/mirna_entry.pl?acc=MI0000071) | -0,626589 | 0,003885792 |
| hsa-miR-335-5p | [MI0000816](https://www.mirbase.org/cgi-bin/mirna_entry.pl?acc=MI0000816) | -0,430071 | 0,003885792 |
| hsa-miR-375-3p | [MI0000783](https://www.mirbase.org/cgi-bin/mirna_entry.pl?acc=MI0000783) | -0,977617 | 0,005510702 |
| hsa-miR-29b-3p | [MI0000105](https://www.mirbase.org/cgi-bin/mirna_entry.pl?acc=MI0000105) | 0,601795 | 0,008900972 |
| hsa-miR-194-5p | [MI0000488](https://www.mirbase.org/cgi-bin/mirna_entry.pl?acc=MI0000488) | 1,184724 | 0,010269111 |
| hsa-miR-144-5p | [MI0000460](https://www.mirbase.org/cgi-bin/mirna_entry.pl?acc=MI0000460) | 0,61031 | 0,010269111 |
| hsa-miR-92b-3p | [MI0003560](https://www.mirbase.org/cgi-bin/mirna_entry.pl?acc=MI0003560) | -0,563217 | 0,010508729 |
| hsa-miR-192-5p | [MI0000234](https://www.mirbase.org/cgi-bin/mirna_entry.pl?acc=MI0000234) | 0,737232 | 0,010713595 |
| hsa-miR-190b-5p | [MI0005545](https://www.mirbase.org/cgi-bin/mirna_entry.pl?acc=MI0005545) | 0,911847 | 0,011519005 |
| hsa-let-7d-5p | [MI0000065](https://www.mirbase.org/cgi-bin/mirna_entry.pl?acc=MI0000065) | -0,336479 | 0,01172507 |
| hsa-miR-140-3p | [MI0000456](https://www.mirbase.org/cgi-bin/mirna_entry.pl?acc=MI0000456) | -0,516976 | 0,012201 |
| hsa-miR-486-3p | [MI0002470](https://www.mirbase.org/cgi-bin/mirna_entry.pl?acc=MI0002470) | -0,594526 | 0,014620297 |
| hsa-miR-190a-5p | [MI0000486](https://www.mirbase.org/cgi-bin/mirna_entry.pl?acc=MI0000486) | 0,566284 | 0,014620297 |
| hsa-miR-30c-5p | [MI0000254](https://www.mirbase.org/cgi-bin/mirna_entry.pl?acc=MI0000254) | 0,465098 | 0,015402122 |
| hsa-miR-128-3p | [MI0000447](https://www.mirbase.org/cgi-bin/mirna_entry.pl?acc=MI0000447) | -0,380273 | 0,016134113 |
| hsa-miR-23a-3p | [MI0000079](https://www.mirbase.org/cgi-bin/mirna_entry.pl?acc=MI0000079) | 0,274765 | 0,019576054 |
| hsa-miR-532-5p | [MI0003205](https://www.mirbase.org/cgi-bin/mirna_entry.pl?acc=MI0003205) | -0,624263 | 0,020201795 |
| hsa-miR-221-3p | [MI0000298](https://www.mirbase.org/cgi-bin/mirna_entry.pl?acc=MI0000298) | 0,285669 | 0,022395562 |
| hsa-miR-1260b | [MI0014197](https://www.mirbase.org/cgi-bin/mirna_entry.pl?acc=MI0014197) | -0,638325 | 0,022628089 |
| hsa-miR-122-5p | [MI0000442](https://www.mirbase.org/cgi-bin/mirna_entry.pl?acc=MI0000442) | 0,611676 | 0,025196637 |
| hsa-miR-328-3p | [MI0000804](https://www.mirbase.org/cgi-bin/mirna_entry.pl?acc=MI0000804) | -0,458376 | 0,027618264 |
| hsa-miR-99a-5p | [MI0000101](https://www.mirbase.org/cgi-bin/mirna_entry.pl?acc=MI0000101) | 0,639743 | 0,029955056 |
| hsa-miR-26a-5p | [MI0000083](https://www.mirbase.org/cgi-bin/mirna_entry.pl?acc=MI0000083) | 0,21141 | 0,030084903 |
| hsa-miR-574-3p | [MI0003581](https://www.mirbase.org/cgi-bin/mirna_entry.pl?acc=MI0003581) | 0,54683 | 0,031846197 |
| hsa-miR-130a-3p | [MI0000448](https://www.mirbase.org/cgi-bin/mirna_entry.pl?acc=MI0000448) | 0,42598 | 0,035018128 |
| hsa-miR-24-3p | [MI0000080](https://www.mirbase.org/cgi-bin/mirna_entry.pl?acc=MI0000080) | 0,243116 | 0,038441228 |
| hsa-miR-29a-3p | [MI0000087](https://www.mirbase.org/cgi-bin/mirna_entry.pl?acc=MI0000087) | 0,41378 | 0,039874778 |
| hsa-miR-7-5p | [MI0000263](https://www.mirbase.org/cgi-bin/mirna_entry.pl?acc=MI0000263) | -0,483343 | 0,050387244 |
| hsa-miR-127-3p | [MI0000472](https://www.mirbase.org/cgi-bin/mirna_entry.pl?acc=MI0000472) | -0,584976 | 0,050414625 |
| hsa-miR-184 | [MI0000481](https://www.mirbase.org/cgi-bin/mirna_entry.pl?acc=MI0000481) | 0,675437 | 0,059587695 |
| hsa-miR-4433b-3p | [MI0025511](https://www.mirbase.org/cgi-bin/mirna_entry.pl?acc=MI0025511) | -0,537323 | 0,060889771 |
| hsa-miR-27b-3p | [MI0000440](https://www.mirbase.org/cgi-bin/mirna_entry.pl?acc=MI0000440) | -0,337083 | 0,077057265 |
| hsa-miR-144-3p | [MI0000460](https://www.mirbase.org/cgi-bin/mirna_entry.pl?acc=MI0000460) | -0,345824 | 0,085286034 |
| hsa-miR-98-5p | [MI0000100](https://www.mirbase.org/cgi-bin/mirna_entry.pl?acc=MI0000100) | -0,270984 | 0,13107799 |
| hsa-miR-1180-3p | [MI0006273](https://www.mirbase.org/cgi-bin/mirna_entry.pl?acc=MI0006273) | -0,381764 | 0,139075258 |
| hsa-miR-15a-5p | [MI0000069](https://www.mirbase.org/cgi-bin/mirna_entry.pl?acc=MI0000069) | 0,264773 | 0,168144941 |
| hsa-miR-4732-5p | [MI0017369](https://www.mirbase.org/cgi-bin/mirna_entry.pl?acc=MI0017369) | 0,385321 | 0,18184628 |
| hsa-miR-181b-5p | [MI0000270](https://www.mirbase.org/cgi-bin/mirna_entry.pl?acc=MI0000270) | -0,288215 | 0,184688355 |
| hsa-miR-625-3p | [MI0003639](https://www.mirbase.org/cgi-bin/mirna_entry.pl?acc=MI0003639) | 0,309563 | 0,186738044 |
| hsa-miR-30a-5p | [MI0000088](https://www.mirbase.org/cgi-bin/mirna_entry.pl?acc=MI0000088) | 0,238589 | 0,186738044 |
| hsa-miR-148a-3p | [MI0000253](https://www.mirbase.org/cgi-bin/mirna_entry.pl?acc=MI0000253) | -0,206418 | 0,195218226 |
| hsa-miR-374a-5p | [MI0000782](https://www.mirbase.org/cgi-bin/mirna_entry.pl?acc=MI0000782) | -0,289637 | 0,195218226 |
| hsa-miR-15b-5p | [MI0000438](https://www.mirbase.org/cgi-bin/mirna_entry.pl?acc=MI0000438) | -0,199403 | 0,209889905 |
| hsa-miR-484 | [MI0002468](https://www.mirbase.org/cgi-bin/mirna_entry.pl?acc=MI0002468) | 0,295256 | 0,213354117 |
| hsa-miR-155-5p | [MI0000681](https://www.mirbase.org/cgi-bin/mirna_entry.pl?acc=MI0000681) | 0,21931 | 0,218730867 |
| hsa-miR-10a-5p | [MI0000266](https://www.mirbase.org/cgi-bin/mirna_entry.pl?acc=MI0000266) | 0,215991 | 0,227382212 |
| hsa-miR-339-5p | [MI0000815](https://www.mirbase.org/cgi-bin/mirna_entry.pl?acc=MI0000815) | 0,256033 | 0,227382212 |
| hsa-miR-584-5p | [MI0003591](https://www.mirbase.org/cgi-bin/mirna_entry.pl?acc=MI0003591) | -0,208258 | 0,227382212 |
| hsa-miR-107 | [MI0000114](https://www.mirbase.org/cgi-bin/mirna_entry.pl?acc=MI0000114) | -0,232468 | 0,228595746 |
| hsa-let-7f-5p | [MI0000067](https://www.mirbase.org/cgi-bin/mirna_entry.pl?acc=MI0000067) | 0,144056 | 0,228595746 |
| hsa-miR-191-5p | [MI0000465](https://www.mirbase.org/cgi-bin/mirna_entry.pl?acc=MI0000465) | -0,157991 | 0,24589208 |
| hsa-miR-27a-3p | [MI0000085](https://www.mirbase.org/cgi-bin/mirna_entry.pl?acc=MI0000085) | -0,184055 | 0,24589208 |
| hsa-miR-1301-3p | [MI0003815](https://www.mirbase.org/cgi-bin/mirna_entry.pl?acc=MI0003815) | 0,297866 | 0,267468947 |
| hsa-miR-532-3p | [MI0003205](https://www.mirbase.org/cgi-bin/mirna_entry.pl?acc=MI0003205) | 0,346302 | 0,279423771 |
| hsa-miR-941 | [MI0005763](https://www.mirbase.org/cgi-bin/mirna_entry.pl?acc=MI0005763) | -0,277139 | 0,313829298 |
| hsa-miR-126-5p | [MI0000471](https://www.mirbase.org/cgi-bin/mirna_entry.pl?acc=MI0000471) | -0,122709 | 0,357389012 |
| hsa-miR-2110 | [MI0010629](https://www.mirbase.org/cgi-bin/mirna_entry.pl?acc=MI0010629) | -0,211211 | 0,384830544 |
| hsa-miR-1307-3p | [MI0006444](https://www.mirbase.org/cgi-bin/mirna_entry.pl?acc=MI0006444) | 0,155507 | 0,390661763 |
| hsa-miR-660-5p | [MI0003684](https://www.mirbase.org/cgi-bin/mirna_entry.pl?acc=MI0003684) | 0,209315 | 0,422750798 |
| hsa-let-7a-5p | [MI0000060](https://www.mirbase.org/cgi-bin/mirna_entry.pl?acc=MI0000060) | -0,093857 | 0,426322282 |
| hsa-miR-10b-5p | [MI0000267](https://www.mirbase.org/cgi-bin/mirna_entry.pl?acc=MI0000267) | 0,150372 | 0,430098574 |
| hsa-miR-183-5p | [MI0000273](https://www.mirbase.org/cgi-bin/mirna_entry.pl?acc=MI0000273) | -0,194964 | 0,48133091 |
| hsa-miR-361-3p | [MI0000760](https://www.mirbase.org/cgi-bin/mirna_entry.pl?acc=MI0000760) | -0,146222 | 0,506743839 |
| hsa-miR-143-3p | [MI0000459](https://www.mirbase.org/cgi-bin/mirna_entry.pl?acc=MI0000459) | -0,215617 | 0,506743839 |
| hsa-miR-224-5p | [MI0000301](https://www.mirbase.org/cgi-bin/mirna_entry.pl?acc=MI0000301) | 0,15869 | 0,529378316 |
| hsa-miR-26b-5p | [MI0000084](https://www.mirbase.org/cgi-bin/mirna_entry.pl?acc=MI0000084) | -0,059696 | 0,570751091 |
| hsa-miR-134-5p | [MI0000474](https://www.mirbase.org/cgi-bin/mirna_entry.pl?acc=MI0000474) | -0,130046 | 0,608657178 |
| hsa-miR-206 | [MI0000490](https://www.mirbase.org/cgi-bin/mirna_entry.pl?acc=MI0000490) | 0,245085 | 0,645397664 |
| hsa-miR-125a-5p | [MI0000469](https://www.mirbase.org/cgi-bin/mirna_entry.pl?acc=MI0000469) | 0,043029 | 0,783325584 |
| hsa-miR-146b-5p | [MI0003129](https://www.mirbase.org/cgi-bin/mirna_entry.pl?acc=MI0003129) | 0,056496 | 0,794078645 |
| hsa-miR-423-3p | [MI0001445](https://www.mirbase.org/cgi-bin/mirna_entry.pl?acc=MI0001445) | 0,04893 | 0,81031412 |
| hsa-miR-483-3p | [MI0002467](https://www.mirbase.org/cgi-bin/mirna_entry.pl?acc=MI0002467) | 0,04962 | 0,892926067 |
| hsa-miR-99b-5p | [MI0000746](https://www.mirbase.org/cgi-bin/mirna_entry.pl?acc=MI0000746) | -0,022878 | 0,908498243 |
| hsa-miR-182-5p | [MI0000272](https://www.mirbase.org/cgi-bin/mirna_entry.pl?acc=MI0000272) | -0,035785 | 0,908498243 |
| hsa-miR-100-5p | [MI0000102](https://www.mirbase.org/cgi-bin/mirna_entry.pl?acc=MI0000102) | 0,04794 | 0,915228673 |
| hsa-miR-1908-5p | [MI0008329](https://www.mirbase.org/cgi-bin/mirna_entry.pl?acc=MI0008329) | -0,007757 | 0,986873788 |
| hsa-miR-152-3p | [MI0000462](https://www.mirbase.org/cgi-bin/mirna_entry.pl?acc=MI0000462) | -0,000108 | 0,999507947 |
| hsa-miR-744-5p | [MI0005559](https://www.mirbase.org/cgi-bin/mirna_entry.pl?acc=MI0005559) | 0,001007 | 0,999507947 |
