# Supplementary material for: Circulating microRNA signatures associated with disease severity and outcome in COVID-19 patients
Source: Front Immunol. 2022 Aug 11;13:968991. doi: 10.3389/fimmu.2022.968991 (PMC9403711; doi:10.3389/fimmu.2022.968991)
Supplement: Supplementary file 5 [file Table_2.docx]

**Supplementary Table 2.** Differentially expressed serum isomiRs in COVID-19 patients vs. healthy controls.

| **IsomiR** | **sequence** | **miRBase** | **miRNA** | **exp_qrt** | **log2FC** | **pvalue** | **padj** |
| --- | --- | --- | --- | --- | --- | --- | --- |
| iso-21-8YUYFYKSB | TCCTGTACTGAGCTGCCCCGA | MI0023622\|MI0002470 | hsa-mir-486-2\|hsa-mir-486-1 | Q3_Max | -1,82 | 3,46E-78 | 5,71E-76 |
| iso-22-8YUYFYKSH | TCCTGTACTGAGCTGCCCCGAG | MI0023622\|MI0002470 | hsa-mir-486-2\|hsa-mir-486-1 | Q3_Max | -1,99 | 5,49E-75 | 4,53E-73 |
| iso-20-3WEUQVKU | CCTGTACTGAGCTGCCCCGA | MI0023622\|MI0002470 | hsa-mir-486-2\|hsa-mir-486-1 | Q3_Max | -1,85 | 1,09E-70 | 6,02E-69 |
| iso-19-FXJYWVJY | AGCTACATTGTCTGCTGGG | MI0000298 | hsa-mir-221 | Mdn_Q3 | 3,08 | 4,11E-58 | 1,69E-55 |
| iso-19-B0NKZ0EY | AAAAGCTGGGTTGAGAGGG | MI0000542\|MI0003776\|MI0003778\|MI0008191\|MI0003839 | hsa-mir-320a\|hsa-mir-320b-1\|hsa-mir-320c-1\|hsa-mir-320c-2\|hsa-mir-320b-2 | Mdn_Q3 | 3,65 | 1,31E-56 | 4,33E-55 |
| iso-20-B0NKZ01J | AAAAGCTGGGTTGAGAGGGC | MI0000542\|MI0003776\|MI0003839 | hsa-mir-320a\|hsa-mir-320b-1\|hsa-mir-320b-2 | Q3_Max | 2,7 | 2,96E-55 | 8,13E-54 |
| iso-21-VFPHUEI8D | TAGCAGCACGTAAATATTGGC | MI0000070\|MI0000115 | hsa-mir-16-1\|hsa-mir-16-2 | Q3_Max | -1,44 | 5,11E-54 | 1,20E-52 |
| iso-21-JY2ZS9R70 | CATTGCACTTGTCTCGGTCTG | MI0000082 | hsa-mir-25 | Mdn_Q3 | -1,53 | 1,78E-51 | 3,66E-51 |
| iso-21-DDR0O0DKB | AAGACGGGAGGAAAGAAGGGA | MI0002467 | hsa-mir-483 | Q3_Max | 3,84 | 3,88E-48 | 7,11E-47 |
| iso-21-B0NKZ01J0 | AAAAGCTGGGTTGAGAGGGCG | MI0000542 | hsa-mir-320a | Q3_Max | 2,32 | 2,42E-44 | 4,00E-43 |
| iso-18-H5IVJKDE | ATCACATTGCCAGGGATT | MI0000079\|MI0000439 | hsa-mir-23a\|hsa-mir-23b | Q1_Mdn | 2,75 | 4,66E-45 | 7,00E-43 |
| iso-21-3WEUQVKU0 | CCTGTACTGAGCTGCCCCGAG | MI0023622\|MI0002470 | hsa-mir-486-2\|hsa-mir-486-1 | Mdn_Q3 | -2,01 | 2,87E-41 | 3,95E-40 |
| iso-20-FXJYWV93 | AGCTACATTGTCTGCTGGGT | MI0000298 | hsa-mir-221 | Mdn_Q3 | 2,35 | 4,30E-39 | 5,46E-38 |
| iso-20-XK6PFJ5D | TGAGGGGCAGAGAGCGAGAC | MI0001445 | hsa-mir-423 | Q3_Max | 1,86 | 4,99E-38 | 5,88E-37 |
| iso-20-FP2WBLZJ | AGCAGCACGTAAATATTGGC | MI0000070\|MI0000115 | hsa-mir-16-1\|hsa-mir-16-2 | Mdn_Q3 | -1,41 | 9,50E-36 | 1,04E-35 |
| iso-20-VIV6OYIN | TAGCTTATCAGACTGATGTT | MI0000077 | hsa-mir-21 | Q3_Max | 2,13 | 3,29E-34 | 3,40E-33 |
| iso-22-20XYYWPKO | CAAAGTGCTGTTCGTGCAGGTA | MI0000095 | hsa-mir-93 | Mdn_Q3 | -1,1 | 7,59E-33 | 7,37E-32 |
| iso-19-XK6PFJHX | TGAGGGGCAGAGAGCGAGA | MI0001445 | hsa-mir-423 | Mdn_Q3 | 2,82 | 3,15E-31 | 2,89E-30 |
| iso-21-UPUYXLEUB | TACAGTACTGTGATAACTGAA | MI0000103\|MI0000739 | hsa-mir-101-1\|hsa-mir-101-2 | Mdn_Q3 | -1,7 | 1,16E-29 | 1,01E-28 |
| iso-21-B175JXN0E | AAACCGTTACCATTACTGAGT | MI0001729 | hsa-mir-451a | Mdn_Q3 | -1,92 | 3,89E-29 | 3,21E-28 |
| iso-21-XK6PFJ5DE | TGAGGGGCAGAGAGCGAGACT | MI0001445 | hsa-mir-423 | Q3_Max | 1,61 | 4,37E-25 | 3,43E-24 |
| iso-21-XKVLRYVPE | TGAGGTAGTAGGTTGTATAGT | MI0000061\|MI0000060\|MI0000062 | hsa-let-7a-2\|hsa-let-7a-1\|hsa-let-7a-3 | Q3_Max | 0,98 | 6,01E-22 | 4,50E-21 |
| iso-22-B175JXN0Q | AAACCGTTACCATTACTGAGTT | MI0001729 | hsa-mir-451a | Mdn_Q3 | -1,66 | 3,07E-20 | 2,20E-19 |
| iso-19-XKVLRYJB | TGAGGTAGTAGGTTGTATA | MI0000061\|MI0000060\|MI0000062 | hsa-let-7a-2\|hsa-let-7a-1\|hsa-let-7a-3 | Q3_Max | -1,1 | 1,15E-19 | 7,93E-19 |
| iso-22-XK6PFJ5DQ | TGAGGGGCAGAGAGCGAGACTT | MI0001445 | hsa-mir-423 | Q3_Max | 1,27 | 5,99E-18 | 3,95E-17 |
| iso-23-9O0MKUN29 | TGTAAACATCCCCGACTGGAAGC | MI0000255 | hsa-mir-30d | Mdn_Q3 | -0,96 | 1,58E-17 | 1,00E-16 |
| iso-20-VFPHUEI8 | TAGCAGCACGTAAATATTGG | MI0000070\|MI0000115 | hsa-mir-16-1\|hsa-mir-16-2 | Mdn_Q3 | -1,08 | 4,22E-17 | 2,58E-16 |
| iso-21-DU2U8IKWE | AATGACACGATCACTCCCGTT | MI0001448 | hsa-mir-425 | min_Q1 | -1,78 | 9,50E-17 | 5,60E-16 |
| iso-19-5KP25HER | GAGGGGCAGAGAGCGAGAC | MI0001445 | hsa-mir-423 | Q1_Mdn | 1,95 | 2,14E-15 | 1,22E-14 |
| iso-21-VY2ZSR670 | TATTGCACTTGTCCCGGCCTG | MI0000094\|MI0000093 | hsa-mir-92a-2\|hsa-mir-92a-1 | Q3_Max | -0,93 | 1,16E-14 | 6,35E-14 |
| iso-19-B175JXHL | AAACCGTTACCATTACTGA | MI0001729 | hsa-mir-451a | Q3_Max | -1,44 | 2,13E-14 | 1,13E-13 |
| iso-20-XKVL7YXY | TGAGGTAGTAGTTTGTGCTG | MI0000434 | hsa-let-7i | Q3_Max | -0,92 | 3,28E-14 | 1,69E-13 |
| iso-21-YOSEKKHKD | TTCAAGTAATCCAGGATAGGC | MI0000750\|MI0000083 | hsa-mir-26a-2\|hsa-mir-26a-1 | Q3_Max | 0,86 | 1,04E-13 | 5,21E-13 |
| iso-21-W7MI3SMID | TCTCTCGGCTCCTCGCGGCTC | MI0016005 | hsa-mir-3615 | Mdn_Q3 | 1,45 | 2,38E-13 | 1,15E-12 |
| iso-22-FPJYUP6XP | AGCAGCATTGTACAGGGCTATG | MI0000108\|MI0000109 | hsa-mir-103a-2\|hsa-mir-103a-1 | Q3_Max | 0,96 | 6,42E-11 | 2,86E-11 |
| iso-21-XKVL5YVPE | TGAGGTAGTAGATTGTATAGT | MI0000067\|MI0000068 | hsa-let-7f-1\|hsa-let-7f-2 | Q3_Max | 0,91 | 4,20E-11 | 1,98E-10 |
| iso-21-WL341OU4D | TCGTACCGTGAGTAATAATGC | MI0000471 | hsa-mir-126 | Q3_Max | 0,97 | 4,65E-11 | 2,13E-10 |
| iso-21-9P9Z35ZQ0 | TGTAGTGTTTCCTACTTTATG | MI0000458 | hsa-mir-142 | Q3_Max | 0,98 | 1,41E-10 | 6,12E-10 |
| iso-21-5KP25HFIE | GAGGGGCAGAGAGCGAGACTT | MI0001445 | hsa-mir-423 | Q1_Mdn | 1,32 | 4,45E-09 | 1,75E-09 |
| iso-19-DIPPZBHR | AAGCTGCCAGTTGAAGAAC | MI0000078 | hsa-mir-22 | min_Q1 | 2,11 | 5,27E-10 | 2,23E-09 |
| iso-22-DU2U8IKWP | AATGACACGATCACTCCCGTTG | MI0001448 | hsa-mir-425 | Q1_Mdn | -1,51 | 5,86E-10 | 2,42E-09 |
| iso-21-SPZW2E0RB | GTCAGTTTGTCAAATACCCCA | MI0000300 | hsa-mir-223 | Mdn_Q3 | -1,32 | 2,61E-08 | 9,56E-09 |
| iso-22-9O0MKUN2H | TGTAAACATCCCCGACTGGAAG | MI0000255 | hsa-mir-30d | Q1_Mdn | -1,49 | 3,61E-10 | 1,45E-08 |
| iso-21-DIPPZBOI0 | AAGCTGCCAGTTGAAGAACTG | MI0000078 | hsa-mir-22 | Q1_Mdn | 1,48 | 7,81E-09 | 3,00E-08 |
| iso-19-XKVL5YJB | TGAGGTAGTAGATTGTATA | MI0000067\|MI0000068 | hsa-let-7f-1\|hsa-let-7f-2 | Mdn_Q3 | -1,08 | 9,54E-09 | 3,58E-08 |
| iso-21-90FB6PY70 | TGGAGAGAAAGGCAGTTCCTG | MI0000482 | hsa-mir-185 | min_Q1 | -1,25 | 1,11E-07 | 3,99E-07 |
| iso-20-9O0MKUN2 | TGTAAACATCCCCGACTGGA | MI0000255 | hsa-mir-30d | min_Q1 | -1,49 | 1,96E-06 | 6,89E-06 |
| iso-22-VIV6OYINL | TAGCTTATCAGACTGATGTTGA | MI0000077 | hsa-mir-21 | Mdn_Q3 | 1 | 2,36E-06 | 8,10E-06 |
| iso-20-90FB6PY7 | TGGAGAGAAAGGCAGTTCCT | MI0000482 | hsa-mir-185 | min_Q1 | -1,37 | 3,18E-06 | 9,89E-06 |
| iso-20-B175JXN0 | AAACCGTTACCATTACTGAG | MI0001729 | hsa-mir-451a | Mdn_Q3 | -1,27 | 5,81E-06 | 1,96E-05 |
| iso-20-XKVLRY98 | TGAGGTAGTAGGTTGTGTGG | MI0000063 | hsa-let-7b | Q3_Max | -0,84 | 7,17E-06 | 2,37E-05 |
| iso-18-JFMOHND9 | CAGCACGTAAATATTGGC | MI0000070\|MI0000115 | hsa-mir-16-1\|hsa-mir-16-2 | min_Q1 | -1,38 | 1,34E-05 | 4,33E-05 |
| iso-19-XKVL7YJW | TGAGGTAGTAGTTTGTGCT | MI0000434 | hsa-let-7i | Mdn_Q3 | -0,95 | 2,79E-05 | 8,84E-05 |
| iso-21-XKV2RYVPE | TGAGGTAGGAGGTTGTATAGT | MI0000066 | hsa-let-7e | Q1_Mdn | 1,58 | 6,53E-05 | 2,00E-04 |
| iso-22-U0XZH3PKO | TAAAGTGCTTATAGTGCAGGTA | MI0000076 | hsa-mir-20a | Q1_Mdn | -1,13 | 1,69E-03 | 4,97E-04 |
| iso-22-967Y8BUR2 | TGTCAGTTTGTCAAATACCCCA | MI0000300 | hsa-mir-223 | Q3_Max | -1 | 7,33E-04 | 2,20E-03 |
| iso-18-FPJYUPD9 | AGCAGCATTGTACAGGGC | MI0000108\|MI0000109\|MI0000114 | hsa-mir-103a-2\|hsa-mir-103a-1\|hsa-mir-107 | Mdn_Q3 | 1,12 | 4,17E-02 | 1,13E-02 |
| iso-20-JY2ZS9R7 | CATTGCACTTGTCTCGGTCT | MI0000082 | hsa-mir-25 | min_Q1 | -1,24 | 8,96E-03 | 2,59E-02 |
| iso-20-531PIWH3 | GAGGTAGTAGATTGTATAGT | MI0000067\|MI0000068 | hsa-let-7f-1\|hsa-let-7f-2 | min_Q1 | 1,18 | 1,31E-02 | 3,73E-02 |
| iso-23-8YUYFYKSX | TCCTGTACTGAGCTGCCCCGAGA | MI0023622\|MI0002470 | hsa-mir-486-2\|hsa-mir-486-1 | Q1_Mdn | -1,19 | 2,64E-02 | 7,37E-02 |
| iso-21-83PM02EZ0 | TCAGTGCATCACAGAACTTTG | MI0000811 | hsa-mir-148b | Q1_Mdn | -1,02 | 3,77E-02 | 1,04E-01 |
| iso-22-XKVLRYVPQ | TGAGGTAGTAGGTTGTATAGTT | MI0000061\|MI0000060\|MI0000062 | hsa-let-7a-2\|hsa-let-7a-1\|hsa-let-7a-3 | Q1_Mdn | 1 | 7,87E-02 | 2,09E-01 |
| iso-20-BQ8DQWM4 | AACATTCAACGCTGTCGGTG | MI0000269\|MI0000289 | hsa-mir-181a-2\|hsa-mir-181a-1 | Q1_Mdn | -1,42 | 3,12E-01 | 8,17E-01 |
| iso-21-YOSE8KHKE | TTCAAGTAATTCAGGATAGGT | MI0000084 | hsa-mir-26b | Mdn_Q3 | 0,66 | 9,35E-01 | 2,41E+00 |
| iso-19-H5IVJKFQ | ATCACATTGCCAGGGATTT | MI0000079 | hsa-mir-23a | Q1_Mdn | 1,27 | 1,88E-01 | 4,76E+00 |
| iso-21-XKVL7YUPE | TGAGGTAGTAGTTTGTACAGT | MI0000433 | hsa-let-7g | Mdn_Q3 | 0,73 | 2,90E+00 | 7,24E+00 |
| iso-21-FKVLRYJPE | AGAGGTAGTAGGTTGCATAGT | MI0000065 | hsa-let-7d | min_Q1 | 1,1 | 2,95E+00 | 7,26E+00 |
| iso-20-VY2ZSR67 | TATTGCACTTGTCCCGGCCT | MI0000094\|MI0000093 | hsa-mir-92a-2\|hsa-mir-92a-1 | Q3_Max | -0,72 | 4,34E+01 | 9,80E+00 |
| iso-20-531P7WH3 | GAGGTAGTAGGTTGTATAGT | MI0000061\|MI0000060\|MI0000062 | hsa-let-7a-2\|hsa-let-7a-1\|hsa-let-7a-3 | Q1_Mdn | 0,95 | 4,61E+00 | 1,12E+01 |
| iso-20-XKVLRYVP | TGAGGTAGTAGGTTGTATAG | MI0000061\|MI0000060\|MI0000062 | hsa-let-7a-2\|hsa-let-7a-1\|hsa-let-7a-3 | Q3_Max | 0,66 | 4,75E+00 | 1,14E+01 |
| iso-22-UPUYXLEUH | TACAGTACTGTGATAACTGAAG | MI0000103\|MI0000739 | hsa-mir-101-1\|hsa-mir-101-2 | min_Q1 | -1,52 | 5,10E+00 | 1,20E+01 |
| iso-22-8YUYFYKSF | TCCTGTACTGAGCTGCCCCGAA | MI0023622\|MI0002470 | hsa-mir-486-2\|hsa-mir-486-1 | min_Q1 | -1,33 | 1,69E+01 | 3,92E+01 |
| iso-18-9O0MKUDP | TGTAAACATCCCCGACTG | MI0000255 | hsa-mir-30d | Q1_Mdn | 1,17 | 2,26E+01 | 5,17E+01 |
| iso-22-W62PBMP1K | TCTCACACAGAAATCGCACCCG | MI0000805 | hsa-mir-342 | Q1_Mdn | -1,14 | 7,30E+01 | 1,63E+02 |
| iso-19-875D3OHP | TCCCTGAGACCCTAACTTG | MI0000446\|MI0000470 | hsa-mir-125b-1\|hsa-mir-125b-2 | Q1_Mdn | 1,55 | 2,87E+02 | 6,31E+02 |
| iso-21-FPJYUP6XE | AGCAGCATTGTACAGGGCTAT | MI0000108\|MI0000109\|MI0000114 | hsa-mir-103a-2\|hsa-mir-103a-1\|hsa-mir-107 | Mdn_Q3 | 0,75 | 4,26E+02 | 9,26E+02 |
| iso-20-M5MUSEDV | CGTACCGTGAGTAATAATGC | MI0000471 | hsa-mir-126 | Q3_Max | 0,64 | 7,35E+02 | 1,57E+03 |
| iso-22-XKVL5YVPQ | TGAGGTAGTAGATTGTATAGTT | MI0000067\|MI0000068 | hsa-let-7f-1\|hsa-let-7f-2 | Q1_Mdn | 0,81 | 1,84E+03 | 3,89E+03 |
| iso-22-VFPHUEI8K | TAGCAGCACGTAAATATTGGCG | MI0000070\|MI0000115 | hsa-mir-16-1\|hsa-mir-16-2 | Mdn_Q3 | -0,52 | 1,24E+05 | 2,52E+04 |
| iso-20-8K4P8R8S | TCAGGCTCAGTCCCCTCCCG | MI0002468 | hsa-mir-484 | Q3_Max | 0,93 | 1,26E+04 | 2,63E+04 |
| iso-21-2HOMKBFPD | CAACGGAATCCCAAAAGCAGC | MI0000465 | hsa-mir-191 | Q1_Mdn | 0,77 | 1,50E+04 | 3,10E+04 |
| iso-21-9O0MKUN2B | TGTAAACATCCCCGACTGGAA | MI0000255 | hsa-mir-30d | Q1_Mdn | -0,69 | 1,52E+06 | 2,88E+05 |
| iso-20-2HOMKBFP | CAACGGAATCCCAAAAGCAG | MI0000465 | hsa-mir-191 | min_Q1 | -1,05 | 2,96E+05 | 5,95E+05 |
| iso-20-UPUYXLEU | TACAGTACTGTGATAACTGA | MI0000103\|MI0000739 | hsa-mir-101-1\|hsa-mir-101-2 | min_Q1 | -1,27 | 5,00E+05 | 9,95E+05 |
| iso-22-875D3ZB7N | TCCCTGAGACCCTTTAACCTGT | MI0000469 | hsa-mir-125a | Q1_Mdn | 0,82 | 6,28E+05 | 1,23E+06 |
| iso-20-IVEYWSSY | ATTGCACTTGTCTCGGTCTG | MI0000082 | hsa-mir-25 | min_Q1 | -1,25 | 8,64E+05 | 1,68E+06 |
| iso-23-875D3ZB700 | TCCCTGAGACCCTTTAACCTGTG | MI0000469 | hsa-mir-125a | Q1_Mdn | 0,75 | 1,07E+05 | 2,06E+06 |
| iso-21-83PI02EZ0 | TCAGTGCACTACAGAACTTTG | MI0000253 | hsa-mir-148a | Q1_Mdn | -0,78 | 1,70E+06 | 3,19E+06 |
| iso-22-B0NKZ01JL | AAAAGCTGGGTTGAGAGGGCGA | MI0000542 | hsa-mir-320a | Mdn_Q3 | 0,74 | 7,42E+06 | 1,38E+07 |
| iso-19-FKVLRY1B | AGAGGTAGTAGGTTGCATA | MI0000065 | hsa-let-7d | Q1_Mdn | -0,59 | 8,34E+06 | 1,53E+07 |
| iso-23-8YUYFYKSZ | TCCTGTACTGAGCTGCCCCGAGT | MI0023622\|MI0002470 | hsa-mir-486-2\|hsa-mir-486-1 | min_Q1 | -0,82 | 2,43E+07 | 4,41E+07 |
| iso-21-875D3ONW0 | TCCCTGAGACCCTAACTTGTG | MI0000446\|MI0000470 | hsa-mir-125b-1\|hsa-mir-125b-2 | Mdn_Q3 | 1,26 | 5,81E+07 | 1,04E+08 |
| iso-20-XKVL5YVP | TGAGGTAGTAGATTGTATAG | MI0000067\|MI0000068 | hsa-let-7f-1\|hsa-let-7f-2 | Q3_Max | 0,45 | 1,64E+08 | 2,91E+08 |
| iso-20-20XYYWPK | CAAAGTGCTGTTCGTGCAGG | MI0000095 | hsa-mir-93 | Q1_Mdn | -0,72 | 1,96E+08 | 3,44E+08 |
| iso-20-XKVL7YUP | TGAGGTAGTAGTTTGTACAG | MI0000433 | hsa-let-7g | Mdn_Q3 | -0,52 | 2,86E+08 | 4,96E+08 |
| iso-20-9OJFERI4 | TGTAACAGCAACTCCATGTG | MI0000732\|MI0000488 | hsa-mir-194-2\|hsa-mir-194-1 | min_Q1 | -1,13 | 7,47E+07 | 1,28E+09 |
| iso-22-9IJNJF6DH | TGGCTCAGTTCAGCAGGAACAG | MI0000081\|MI0000080 | hsa-mir-24-2\|hsa-mir-24-1 | min_Q1 | 0,53 | 8,72E+08 | 1,48E+09 |
| iso-20-967Y8BUR | TGTCAGTTTGTCAAATACCC | MI0000300 | hsa-mir-223 | Mdn_Q3 | -0,51 | 1,03E+09 | 1,74E+09 |
| iso-19-X2X02Y29 | TGAGATGAAGCACTGTAGC | MI0000459 | hsa-mir-143 | min_Q1 | -0,87 | 1,09E+09 | 1,81E+09 |
| iso-20-83PI02EZ | TCAGTGCACTACAGAACTTT | MI0000253 | hsa-mir-148a | Mdn_Q3 | 0,63 | 1,54E+09 | 2,54E+09 |
| iso-20-IVEY8SPY | ATTGCACTTGTCCCGGCCTG | MI0000094\|MI0000093 | hsa-mir-92a-2\|hsa-mir-92a-1 | Q3_Max | -0,39 | 2,88E+07 | 4,70E+09 |
| iso-20-JXYIZ8UV | CATTATTACTTTTGGTACGC | MI0000471 | hsa-mir-126 | Q3_Max | 0,39 | 5,27E+09 | 8,53E+09 |
| iso-23-X2EUIRIK0P | TGAGAACTGAATTCCATGGGTTG | MI0000477 | hsa-mir-146a | min_Q1 | -0,64 | 5,63E+09 | 9,02E+09 |
| iso-19-VFPHUEFP | TAGCAGCACGTAAATATTG | MI0000070\|MI0000115 | hsa-mir-16-1\|hsa-mir-16-2 | Q3_Max | -0,36 | 7,85E+09 | 0,000124 |
| iso-21-967Y8BURD | TGTCAGTTTGTCAAATACCCC | MI0000300 | hsa-mir-223 | Mdn_Q3 | -0,43 | 7,96E+08 | 0,000125 |
| iso-19-2HOMKBEU | CAACGGAATCCCAAAAGCA | MI0000465 | hsa-mir-191 | Q1_Mdn | 0,6 | 8,50E+09 | 0,000132 |
| iso-20-0317PFZ3 | ACAGTAGTCTGCACATTGGT | MI0000281\|MI0000282\|MI0000242 | hsa-mir-199a-2\|hsa-mir-199b\|hsa-mir-199a-1 | Mdn_Q3 | -0,4 | 0.0001255 | 0,000194 |
| iso-22-XKVL7YXYQ | TGAGGTAGTAGTTTGTGCTGTT | MI0000434 | hsa-let-7i | Q1_Mdn | -0,37 | 0.0001510 | 0,000231 |
| iso-22-F9R75JK9H | AGCTCGGTCTGAGGCCCCTCAG | MI0001445 | hsa-mir-423 | Mdn_Q3 | 0,52 | 0.0002409 | 0,000365 |
| iso-22-X2EUIRIKQ | TGAGAACTGAATTCCATGGGTT | MI0000477 | hsa-mir-146a | Q1_Mdn | -0,43 | 0.0003153 | 0,000473 |
| iso-19-XKVLRYK0 | TGAGGTAGTAGGTTGTGTG | MI0000063 | hsa-let-7b | Q3_Max | 0,31 | 0.0004738 | 0,000704 |
| iso-19-8YUYFY1J | TCCTGTACTGAGCTGCCCC | MI0023622\|MI0002470 | hsa-mir-486-2\|hsa-mir-486-1 | Q3_Max | 0,33 | 0.0006331 | 0,000933 |
| iso-23-967Y8BURDF | TGTCAGTTTGTCAAATACCCCAA | MI0000300 | hsa-mir-223 | Mdn_Q3 | 0,43 | 0.0009891 | 0,001444 |
| iso-23-XKVLRY980Q | TGAGGTAGTAGGTTGTGTGGTTT | MI0000063 | hsa-let-7b | Q1_Mdn | 0,38 | 0.0010896 | 0,001577 |
| iso-19-9O0MKUH4 | TGTAAACATCCCCGACTGG | MI0000255 | hsa-mir-30d | Q1_Mdn | -0,49 | 0.0013627 | 0,001955 |
| iso-19-IVEY8SI3 | ATTGCACTTGTCCCGGCCT | MI0000094\|MI0000093 | hsa-mir-92a-2\|hsa-mir-92a-1 | Q1_Mdn | -0,41 | 0.0017758 | 0,002526 |
| iso-21-XKVLRY98E | TGAGGTAGTAGGTTGTGTGGT | MI0000063 | hsa-let-7b | Q3_Max | -0,25 | 0.0022132 | 0,003121 |
| iso-19-YOSEKKFH | TTCAAGTAATCCAGGATAG | MI0000750\|MI0000083 | hsa-mir-26a-2\|hsa-mir-26a-1 | Mdn_Q3 | 0,34 | 0.0027180 | 0,003769 |
| iso-20-VFKNXBW3 | TAGCACCATTTGAAATCGGT | MI0000735 | hsa-mir-29c | min_Q1 | 0,41 | 0.0027118 | 0,003769 |
| iso-22-2HOMKBFP3 | CAACGGAATCCCAAAAGCAGCT | MI0000465 | hsa-mir-191 | Mdn_Q3 | 0,35 | 0.0031426 | 0,004321 |
| iso-21-H5IVJKI9D | ATCACATTGCCAGGGATTTCC | MI0000079 | hsa-mir-23a | min_Q1 | -0,39 | 0.0039106 | 0,005333 |
| iso-19-3WEUQV1K | CCTGTACTGAGCTGCCCCG | MI0023622\|MI0002470 | hsa-mir-486-2\|hsa-mir-486-1 | Mdn_Q3 | -0,32 | 0.0043023 | 0,005819 |
