# Supplementary material for: Circulating microRNA signatures associated with disease severity and outcome in COVID-19 patients
Source: Front Immunol. 2022 Aug 11;13:968991. doi: 10.3389/fimmu.2022.968991 (PMC9403711; doi:10.3389/fimmu.2022.968991)
Supplement: Supplementary file 6 [file Table_3.docx]

**Supplementary Table 3.** Differentially expressed serum isomiRs in severe vs. mild COVID-19

| IsomiR | sequence | miRBase | miRNA | exp_qrt | log2FC | pvalue | padj |
| --- | --- | --- | --- | --- | --- | --- | --- |
| iso-23-XKVLRY980O | TGAGGTAGTAGGTTGTGTGGTTA | MI0000063 | hsa-let-7b | Q1_Mdn | -1,2 | 3,50E+06 | 5,63E+08 |
| iso-19-875D3OHP | TCCCTGAGACCCTAACTTG | MI0000446\|MI0000470 | hsa-mir-125b-1\|hsa-mir-125b-2 | Q1_Mdn | 1,81 | 7,14E+06 | 5,74E+08 |
| iso-21-XKVLRYVPE | TGAGGTAGTAGGTTGTATAGT | MI0000061\|MI0000060\|MI0000062 | hsa-let-7a-2\|hsa-let-7a-1\|hsa-let-7a-3 | Q3_Max | -0,64 | 6,44E+08 | 0.000345 |
| iso-20-VIV6OYIN | TAGCTTATCAGACTGATGTT | MI0000077 | hsa-mir-21 | Q3_Max | 1,06 | 8,85E+08 | 0.000356 |
| iso-22-XKVLRY98Q | TGAGGTAGTAGGTTGTGTGGTT | MI0000063 | hsa-let-7b | Q3_Max | -0,76 | 1,46E+09 | 0.000390 |
| iso-22-XKVL5YVPQ | TGAGGTAGTAGATTGTATAGTT | MI0000067\|MI0000068 | hsa-let-7f-1\|hsa-let-7f-2 | Mdn_Q3 | -0,96 | 1,29E+07 | 0.000390 |
| iso-21-XKVL5YVPE | TGAGGTAGTAGATTGTATAGT | MI0000067\|MI0000068 | hsa-let-7f-1\|hsa-let-7f-2 | Q3_Max | -0,66 | 3,80E+09 | 0.000873 |
| iso-20-B175JXN0 | AAACCGTTACCATTACTGAG | MI0001729 | hsa-mir-451a | Mdn_Q3 | 1,07 | 0.00014245 | 0.002866 |
| iso-20-B0NKZ01J | AAAAGCTGGGTTGAGAGGGC | MI0000542\|MI0003776\|MI0003839 | hsa-mir-320a\|hsa-mir-320b-1\|hsa-mir-320b-2 | Q3_Max | 0,95 | 0.00022213 | 0.003676 |
| iso-18-H5IVJKDE | ATCACATTGCCAGGGATT | MI0000079\|MI0000439 | hsa-mir-23a\|hsa-mir-23b | Mdn_Q3 | 0,82 | 0.00025120 | 0.003676 |
| iso-21-XKV2RYVPE | TGAGGTAGGAGGTTGTATAGT | MI0000066 | hsa-let-7e | Q1_Mdn | -1,14 | 0.00024201 | 0.003676 |
| iso-21-DDR0O0DKB | AAGACGGGAGGAAAGAAGGGA | MI0002467 | hsa-mir-483 | Q3_Max | 1,58 | 0.00029067 | 0.003899 |
| iso-23-XKVLRY980Q | TGAGGTAGTAGGTTGTGTGGTTT | MI0000063 | hsa-let-7b | Q1_Mdn | -0,96 | 0.00039646 | 0.004910 |
| iso-21-XKVL7YXYE | TGAGGTAGTAGTTTGTGCTGT | MI0000434 | hsa-let-7i | Q3_Max | -0,54 | 0.00055149 | 0.006320 |
| iso-20-531PIWH3 | GAGGTAGTAGATTGTATAGT | MI0000067\|MI0000068 | hsa-let-7f-1\|hsa-let-7f-2 | Q1_Mdn | -0,89 | 0.00058882 | 0.006320 |
| iso-20-VY2ZSR67 | TATTGCACTTGTCCCGGCCT | MI0000094\|MI0000093 | hsa-mir-92a-2\|hsa-mir-92a-1 | Q3_Max | 0,75 | 0.00067125 | 0.006357 |
| iso-22-X2EUIRIKQ | TGAGAACTGAATTCCATGGGTT | MI0000477 | hsa-mir-146a | Q1_Mdn | -0,92 | 0.00066598 | 0.006357 |
| iso-21-8YUYFYKSB | TCCTGTACTGAGCTGCCCCGA | MI0023622\|MI0002470 | hsa-mir-486-2\|hsa-mir-486-1 | Q3_Max | 0,6 | 0.00082692 | 0.006825 |
| iso-21-9P9Z35ZQ0 | TGTAGTGTTTCCTACTTTATG | MI0000458 | hsa-mir-142 | Q3_Max | -0,52 | 0.00084784 | 0.006825 |
| iso-20-3WEUQVKU | CCTGTACTGAGCTGCCCCGA | MI0023622\|MI0002470 | hsa-mir-486-2\|hsa-mir-486-1 | Mdn_Q3 | 0,72 | 0.00078720 | 0.006825 |
| iso-21-YOSEKKHKD | TTCAAGTAATCCAGGATAGGC | MI0000750\|MI0000083 | hsa-mir-26a-2\|hsa-mir-26a-1 | Q3_Max | -0,56 | 0.00104292 | 0.007632 |
| iso-19-IVEY8SI3 | ATTGCACTTGTCCCGGCCT | MI0000094\|MI0000093 | hsa-mir-92a-2\|hsa-mir-92a-1 | Q1_Mdn | 0,86 | 0.00099924 | 0.007632 |
| iso-20-IVEY8SPY | ATTGCACTTGTCCCGGCCTG | MI0000094\|MI0000093 | hsa-mir-92a-2\|hsa-mir-92a-1 | Q3_Max | 0,57 | 0.00109123 | 0.007638 |
| iso-22-2HOMKBFP3 | CAACGGAATCCCAAAAGCAGCT | MI0000465 | hsa-mir-191 | Mdn_Q3 | -0,76 | 0.00114841 | 0.007703 |
| iso-21-VY2ZSR670 | TATTGCACTTGTCCCGGCCTG | MI0000094\|MI0000093 | hsa-mir-92a-2\|hsa-mir-92a-1 | Q3_Max | 0,5 | 0.00130762 | 0.008421 |
| iso-19-DIPPZBHR | AAGCTGCCAGTTGAAGAAC | MI0000078 | hsa-mir-22 | min_Q1 | 0,93 | 0.00144906 | 0.008973 |
| iso-19-9O0MKUH4 | TGTAAACATCCCCGACTGG | MI0000255 | hsa-mir-30d | Q1_Mdn | 0,84 | 0.00165111 | 0.009493 |
| iso-20-9OJFERI4 | TGTAACAGCAACTCCATGTG | MI0000732\|MI0000488 | hsa-mir-194-2\|hsa-mir-194-1 | min_Q1 | 1,7 | 0.00162374 | 0.009493 |
| iso-22-XKVLRYVPQ | TGAGGTAGTAGGTTGTATAGTT | MI0000061\|MI0000060\|MI0000062 | hsa-let-7a-2\|hsa-let-7a-1\|hsa-let-7a-3 | Mdn_Q3 | -0,76 | 0.00202208 | 0.011226 |
| iso-20-FXJYWV93 | AGCTACATTGTCTGCTGGGT | MI0000298 | hsa-mir-221 | Mdn_Q3 | 0,61 | 0.00364015 | 0.019535 |
| iso-19-B175JXHL | AAACCGTTACCATTACTGA | MI0001729 | hsa-mir-451a | Mdn_Q3 | 0,81 | 0.00401060 | 0.020829 |
| iso-23-XK6PFJ5D0Q | TGAGGGGCAGAGAGCGAGACTTT | MI0001445 | hsa-mir-423 | Q1_Mdn | -0,73 | 0.00461837 | 0.023236 |
| iso-20-X86XRIBP | TGCGGGGCTAGGGCTAACAG | MI0005559 | hsa-mir-744 | min_Q1 | -0,95 | 0.00516271 | 0.025187 |
| iso-23-967Y8BURDI | TGTCAGTTTGTCAAATACCCCAT | MI0000300 | hsa-mir-223 | Q1_Mdn | -0,61 | 0.00541695 | 0.025526 |
| iso-20-JY2ZS9R7 | CATTGCACTTGTCTCGGTCT | MI0000082 | hsa-mir-25 | min_Q1 | 0,94 | 0.00554932 | 0.025526 |
| iso-21-WL341OU4D | TCGTACCGTGAGTAATAATGC | MI0000471 | hsa-mir-126 | Q3_Max | -0,41 | 0.00697390 | 0.030345 |
| iso-23-9O0MKUN29 | TGTAAACATCCCCGACTGGAAGC | MI0000255 | hsa-mir-30d | Mdn_Q3 | -0,52 | 0.00696938 | 0.030345 |
| iso-21-9IJNJF6DB | TGGCTCAGTTCAGCAGGAACA | MI0000080\|MI0000081 | hsa-mir-24-1\|hsa-mir-24-2 | min_Q1 | -0,81 | 0.00795460 | 0.033702 |
| iso-21-B175JXN0E | AAACCGTTACCATTACTGAGT | MI0001729 | hsa-mir-451a | Mdn_Q3 | 0,79 | 0.00831805 | 0.034338 |
| iso-20-8YUYFYKS | TCCTGTACTGAGCTGCCCCG | MI0023622\|MI0002470 | hsa-mir-486-2\|hsa-mir-486-1 | Q3_Max | 0,63 | 0.00899344 | 0.034563 |
| iso-21-B0NKZ01J0 | AAAAGCTGGGTTGAGAGGGCG | MI0000542 | hsa-mir-320a | Q3_Max | 0,61 | 0.00926304 | 0.034563 |
| iso-21-X2EUIRIKE | TGAGAACTGAATTCCATGGGT | MI0000477 | hsa-mir-146a | Q3_Max | -0,49 | 0.00902941 | 0.034563 |
| iso-20-83PI02EZ | TCAGTGCACTACAGAACTTT | MI0000253 | hsa-mir-148a | Mdn_Q3 | 0,68 | 0.00944602 | 0.034563 |
| iso-20-531P7WH3 | GAGGTAGTAGGTTGTATAGT | MI0000061\|MI0000060\|MI0000062 | hsa-let-7a-2\|hsa-let-7a-1\|hsa-let-7a-3 | Q1_Mdn | -0,63 | 0.00865876 | 0.034563 |
| iso-21-0317PFZ3E | ACAGTAGTCTGCACATTGGTT | MI0000281\|MI0000282\|MI0000242 | hsa-mir-199a-2\|hsa-mir-199b\|hsa-mir-199a-1 | Q1_Mdn | -0,69 | 0.01031168 | 0.036892 |
| iso-21-XKVLRY98E | TGAGGTAGTAGGTTGTGTGGT | MI0000063 | hsa-let-7b | Q3_Max | -0,33 | 0.01057360 | 0.037007 |
| iso-19-VFPHUEFP | TAGCAGCACGTAAATATTG | MI0000070\|MI0000115 | hsa-mir-16-1\|hsa-mir-16-2 | Q3_Max | 0,5 | 0.01280441 | 0.042404 |
| iso-22-BQ8DQWM4H | AACATTCAACGCTGTCGGTGAG | MI0000269\|MI0000289 | hsa-mir-181a-2\|hsa-mir-181a-1 | min_Q1 | -0,85 | 0.01290575 | 0.042404 |
| iso-21-UPVMX5I8E | TACAGTAGTCTGCACATTGGT | MI0000281\|MI0000282\|MI0000242 | hsa-mir-199a-2\|hsa-mir-199b\|hsa-mir-199a-1 | min_Q1 | -0,75 | 0.01266791 | 0.042404 |
| iso-23-X2EUIRIK0P | TGAGAACTGAATTCCATGGGTTG | MI0000477 | hsa-mir-146a | min_Q1 | -0,81 | 0.01342693 | 0.043234 |
| iso-19-FXJYWVJY | AGCTACATTGTCTGCTGGG | MI0000298 | hsa-mir-221 | Mdn_Q3 | 0,6 | 0.01378554 | 0.043519 |
| iso-22-FPJYUP6XP | AGCAGCATTGTACAGGGCTATG | MI0000108\|MI0000109 | hsa-mir-103a-2\|hsa-mir-103a-1 | Q3_Max | -0,38 | 0.01515477 | 0.046921 |
| iso-20-JXYIZ8UV | CATTATTACTTTTGGTACGC | MI0000471 | hsa-mir-126 | Q3_Max | -0,43 | 0.01596970 | 0.048511 |
| iso-19-8YUYFY1J | TCCTGTACTGAGCTGCCCC | MI0023622\|MI0002470 | hsa-mir-486-2\|hsa-mir-486-1 | Q3_Max | 0,6 | 0.01640223 | 0.048902 |
| iso-20-XK6PFJ5D | TGAGGGGCAGAGAGCGAGAC | MI0001445 | hsa-mir-423 | Q3_Max | 0,56 | 0.01732919 | 0.048947 |
| iso-23-875D3ZB700 | TCCCTGAGACCCTTTAACCTGTG | MI0000469 | hsa-mir-125a | Q1_Mdn | -0,64 | 0.01716306 | 0.048947 |
| iso-23-2HOMKBFPDP | CAACGGAATCCCAAAAGCAGCTG | MI0000465 | hsa-mir-191 | Q1_Mdn | -0,74 | 0.01732756 | 0.048947 |
