# Supplementary material for: Circulating microRNA signatures associated with disease severity and outcome in COVID-19 patients
Source: Front Immunol. 2022 Aug 11;13:968991. doi: 10.3389/fimmu.2022.968991 (PMC9403711; doi:10.3389/fimmu.2022.968991)
Supplement: Supplementary file 7 [file Table_4.docx]

**Supplementary Table 4.** Pathways predicted as modulated by upregulated miRNAs in COVID-19 vs HC

| **Pathway ID** | ***Pathway name*** | **FDR** |
| --- | --- | --- |
| hsa04218 | *Cellular senescence* | <0.0001 |
| hsa04010 | *MAPK signaling pathway* | <0.0001 |
| WP3303 | *RAC1/PAK1/p38/MMP2 pathway* | <0.0001 |
| hsa04151 | *PI3K-Akt signaling pathway* | <0.0001 |
| hsa04115 | *p53 signaling pathway* | <0.0001 |
| WP2882 | *Nuclear receptors meta-pathway* | <0.0001 |
| WP23 | *B cell receptor signaling pathway* | <0.0001 |
| hsa04350 | *TGF-beta signaling pathway* | <0.0001 |
| WP304 | *Kit receptor signaling pathway* | <0.0001 |
| WP3624 | *Lung fibrosis* | <0.0001 |
| hsa04066 | *HIF-1 signaling pathway* | <0.0001 |
| hsa05130 | *Pathogenic Escherichia coli infection* | <0.0001 |
| WP127 | *IL-5 signaling pathway* | <0.0001 |
| DOID:178 | *Vascular disease* | <0.0001 |
| WP363 | *Wnt signaling pathway* | <0.0001 |
| WP710 | *DNA damage response (only ATM dependent)* | <0.0001 |
| WP3888 | *VEGFA-VEGFR2 signaling pathway* | <0.0001 |
| hsa04060 | *Cytokine-cytokine receptor interaction* | 0.001 |
| DOID:850 | *Lung disease* | 0.0023 |
| hsa04068 | *FoxO signaling pathway* | 0.0028 |
| hsa04915 | *Estrogen signaling pathway* | 0.0032 |
| WP3931 | *Embryonic stem cell pluripotency pathways* | 0.0032 |
| WP4754 | *IL-18 signaling pathway* | 0.0076 |
| WP1982 | *Sterol regulatory element-binding proteins (SREBP) signaling* | 0.0079 |
| WP4884 | *Pathogenesis of SARS-CoV-2 mediated by nsp9-nsp10 complex* | 0.0124 |
| hsa04210 | *Apoptosis* | 0.0267 |
| DOID:0050117 | *Disease by infectious agent* | 0.0285 |
