# Supplementary material for: Circulating microRNA signatures associated with disease severity and outcome in COVID-19 patients
Source: Front Immunol. 2022 Aug 11;13:968991. doi: 10.3389/fimmu.2022.968991 (PMC9403711; doi:10.3389/fimmu.2022.968991)
Supplement: Supplementary file 8 [file Table_5.docx]

**Supplementary Table 5.** Pathways predicted as modulated by downregulated miRNAs in COVID-19 vs HC

| **Pathway ID** | ***Pathway name*** | **FDR** |
| --- | --- | --- |
| hsa04151 | *PI3K-Akt signaling pathway* | <0.0001 |
| hsa04140 | *Autophagy - animal* | <0.0001 |
| Hsa05160/1 | *Hepatitis C &B* | <0.0001 |
| WP710 | *DNA damage response (only ATM dependent)* | <0.0001 |
| hsa04068 | *FoxO signaling pathway* | <0.0001 |
| WP3888 | *VEGFA-VEGFR2 signaling pathway* | <0.0001 |
| hsa04010 | *MAPK signaling pathway* | <0.0001 |
| hsa04210 | *Apoptosis* | <0.0001 |
| WP5039 | *SARS-CoV-2 innate immunity evasion and cell-specific immune response* | <0.0001 |
| WP366 | *TGF-beta signaling pathway* | <0.0001 |
| hsa04933 | *AGE-RAGE signaling pathway in diabetic complications* | <0.0001 |
| hsa04550 | *Signaling pathways regulating pluripotency of stem cells* | <0.0001 |
| hsa04630 | *JAK-STAT signaling pathway* | <0.0001 |
| WP3931 | *Embryonic stem cell pluripotency pathways* | <0.0001 |
| hsa04218 | *Cellular senescence* | <0.0001 |
| WP4754 | *IL-18 signaling pathway* | <0.0001 |
| WP127 | *IL-5 signaling pathway* | <0.0001 |
| WP75 | *Toll-like receptor signaling pathway* | <0.0001 |
| WP399 | *Wnt signaling pathway and pluripotency* | <0.0001 |
| hsa04115 | *p53 signaling pathway* | <0.0001 |
| DOID:850 | *Lung disease* | <0.0001 |
| DOID:178 | *Vascular disease* | 0.0025 |
