# Supplementary material for: Circulating microRNA signatures associated with disease severity and outcome in COVID-19 patients
Source: Front Immunol. 2022 Aug 11;13:968991. doi: 10.3389/fimmu.2022.968991 (PMC9403711; doi:10.3389/fimmu.2022.968991)
Supplement: Supplementary file 10 [file Table_7.docx]

**Supplementary Table 7.** Pathways predicted as modulated by downregulated miRNAs in severe vs mild/moderate COVID-19

| **Pathway ID** | ***Pathway name*** | **FDR** |
| --- | --- | --- |
| hsa04933 | *AGE-RAGE signaling pathway in diabetic complications* | <0.0001 |
| hsa05161 | *Hepatitis B* | <0.0001 |
| WP4754 | *IL-18 signaling pathway* | <0.0001 |
| hsa04657 | *IL-17 signaling pathway* | <0.0001 |
| hsa04668 | *TNF signaling pathway* | <0.0001 |
| WP75 | *Toll-like receptor signaling pathway* | <0.0001 |
| hsa04151 | *PI3K-Akt signaling pathway* | <0.0001 |
| hsa04660 | *T cell receptor signaling pathway* | <0.0001 |
| hsa04218 | *Cellular senescence* | <0.0001 |
| hsa04630 | *JAK-STAT signaling pathway* | <0.0001 |
| WP3888 | *VEGFA-VEGFR2 signaling pathway* | <0.0001 |
| WP2018 | *RANKL/RANK signaling pathway* | <0.0001 |
| hsa04062 | *Chemokine signaling pathway* | <0.0001 |
| DOID:178 | *Vascular disease* | <0.0001 |
| hsa04110 | *Cell cycle* | <0.0001 |
| WP4969 | *RAS and bradykinin pathways in COVID-19* | <0.0001 |
